# Characteristics and outcomes of COVID-19 patients admitted to hospital with and without respiratory symptoms

**DOI:** 10.1016/j.heliyon.2024.e29591

**Published:** 2024-05-04

**Authors:** Barbara Wanjiru Citarella, Christiana Kartsonaki, Elsa D. Ibáñez-Prada, Bronner P. Gonçalves, Joaquin Baruch, Martina Escher, Mark G. Pritchard, Jia Wei, Fred Philippy, Andrew Dagens, Matthew Hall, James Lee, Demetrios James Kutsogiannis, Evert-Jan Wils, Marília Andreia Fernandes, Bharath Kumar Tirupakuzhi Vijayaraghavan, Prasan Kumar Panda, Ignacio Martin-Loeches, Shinichiro Ohshimo, Arie Zainul Fatoni, Peter Horby, Jake Dunning, Jordi Rello, Laura Merson, Amanda Rojek, Michel Vaillant, Piero Olliaro, Luis Felipe Reyes, the ISARIC Clinical Characterisation Group, S.A. Moharam, Sabriya Abdalasalam, Alaa Abdalfattah Abdalhadi, Naana Reyam Abdalla, Walaa Abdalla, Almthani Hamza Abdalrheem, Ashraf Abdalsalam, Saedah Abdeewi, Esraa Hassan Abdelgaum, Mohamed Abdelhalim, Mohammed Abdelkabir, Israa Abdelrahman, Sheryl Ann Abdukahil, Lamees Adil Abdulbaqi, Salaheddin Abdulhamid, Widyan Abdulhamid, Nurul Najmee Abdulkadir, Eman Abdulwahed, Rawad Abdunabi, Ryuzo Abe, Laurent Abel, Ahmed Mohammed Abodina, Amal Abrous, Lara Absil, Kamal Abu Jabal, Nashat Abu Salah, Abdurraouf Abusalama, Tareg Abdallah Abuzaid, Subhash Acharya, Andrew Acker, Elisabeth Adam, Safia Adem, Manuella Ademnou, Francisca Adewhajah, Diana Adrião, Anthony Afum-Adjei Awuah, Melvin Agbogbatey, Saleh Al Ageel, Aya Mustafa Ahmed, Musaab Mohammed Ahmed, Shakeel Ahmed, Zainab Ahmed Alaraji, Abdulrahman Ahmed Elhefnawy Enan, Reham Abdelhamid Ahmed Khalil, Ali Mostafa Ahmed Mohamed Abdelaziz, Kate Ainscough, Eka Airlangga, Tharwat Aisa, Ali Aisha, Bugila Aisha, Ali Ait Hssain, Younes Ait Tamlihat, Takako Akimoto, Ernita Akmal, Chika Akwani, Eman Al Qasim, Ahmed Alajeeli, Ahmed Alali, Razi Alalqam, Aliya Mohammed Alameen, Mohammed Al-Aquily, Zinah A. Alaraji, Khalid Albakry, Safa Albatni, Angela Alberti, Osama Aldabbourosama, Tala Al-dabbous, Amer Aldhalia, Abdulkarim Aldoukali, Senthilkumar Alegesan, Marta Alessi, Beatrice Alex, Kévin Alexandre, Abdulrahman Al-Fares, Asil Alflite, Huda Alfoudri, Qamrah Alhadad, Hoda Salem Alhaddad, Maali Khalid Mohamed Abdalla Alhasan, Ahmad Nabil Alhouri, Hasan Alhouri, Adam Ali, Imran Ali, Maha TagElser Mohammed Ali, Syed Ali Abbas, Yomna Ali Abdelghafar, Naseem Ali Sheikh, Kazali Enagnon Alidjnou, Mahmoud Aljadi, Sarah Aljamal, Mohammed Alkahlout, Akram Alkaseek, Qabas Alkhafajee, Clotilde Allavena, Nathalie Allou, Lana Almasri, Abdulrahman Almjersah, Raja Ahmed Alqandouz, Walaa Alrfaea, Moayad Alrifaee, Rawan Alsaadi, Yousef Al-Saba'a, Entisar Alshareea, Eslam Alshenawy, Aneela Altaf, João Melo Alves, João Alves, Rita Alves, Joana Alves Cabrita, Maria Amaral, Amro Essam Amer, Nur Amira, Amos Amoako Adusei, John Amuasi, Roberto Andini, Claire Andrejak, Andrea Angheben, François Angoulvant, Sophia Ankrah, Séverine Ansart, Sivanesen Anthonidass, Massimo Antonelli, Carlos Alexandre Antunes de Brito, Ardiyan Apriyana, Yaseen Arabi, Irene Aragao, Francisco Arancibia, Carolline Araujo, Antonio Arcadipane, Patrick Archambault, Lukas Arenz, Jean-Benoît Arlet, Christel Arnold-Day, Lovkesh Arora, Rakesh Arora, Elise Artaud-Macari, Diptesh Aryal, Angel Asensio, Elizabeth A. Ashley, Muhammad Ashraf, Muhammad Sheharyar Ashraf, Abir Ben Ashur, Franklin Asiedu-Bekoe, Namra Asif, Mohammad Asim, Grace Assi, Jean Baptiste Assie, Amirul Asyraf, Fouda Atangana, Ahmed Atia, Minahel Atif, Asia Atif Abdelrhman Abdallahrs, Anika Atique, Moad Atlowly, AM Udara Lakshan Attanyake, Johann Auchabie, Hugues Aumaitre, Adrien Auvet, Abdelmalek Awad Ali Mohammed, Eyvind W. Axelsen, Ared Ayad, Ahmed Ayman Hassan Helmi, Laurène Azemar, Mohammed Azizeldin, Cecile Azoulay, Hakeem Babatunde, Benjamin Bach, Delphine Bachelet, Claudine Badr, Roar Bævre-Jensen, Nadia Baig, John Kenneth Baillie, J Kevin Baird, Erica Bak, Agamemnon Bakakos, Nazreen Abu Bakar, Hibah Bileid Bakeer, Ashraf Bakri, Andriy Bal, Mohanaprasanth Balakrishnan, Irene Bandoh, Firouzé Bani-Sadr, Renata Barbalho, Nicholas Yuri Barbosa, Wendy S. Barclay, Saef Umar Barnett, Michaela Barnikel, Helena Barrasa, Cleide Barrigoto, Marie Bartoli, Joaquín Baruch, Romain Basmaci, Muhammad Fadhli Hassin Basri, AbdAlkarim Batool, Denise Battaglini, Jules Bauer, Diego Fernando Bautista Rincon, Denisse Bazan Dow, Abigail Beane, Alexandra Bedossa, Ker Hong Bee, Husna Begum, Sylvie Behilill, Albertus Beishuizen, Aleksandr Beljantsev, David Bellemare, Anna Beltrame, Beatriz Amorim Beltrão, Marine Beluze, Nicolas Benech, Lionel Eric Benjiman, Suzanne Bennett, Luís Bento, Jan-Erik Berdal, Lamis Berdeweel, Delphine Bergeaud, Hazel Bergin, Giulia Bertoli, Lorenzo Bertolino, Simon Bessis, Sybille Bevilcaqua, Karine Bezulier, Amar Bhatt, Krishna Bhavsar, Isabella Bianchi, Claudia Bianco, Sandra Bichoka, Farah Nadiah Bidin, Felwa Bin Humaid, Mohd Nazlin Bin Kamarudin, Muhannud Binnawara, Zeno Bisoffi, Patrick Biston, Laurent Bitker, Mustapha Bittaye, Jonathan Bitton, Pablo Blanco-Schweizer, Catherine Blier, Frank Bloos, Mathieu Blot, Filomena Boccia, Laetitia Bodenes, Debby Bogaert, Anne-Hélène Boivin, Ariel Bolanga, Isabela Bolaños, Pierre-Adrien Bolze, François Bompart, Aurelius Bonifasius, Joe Bonney, Diogo Borges, Raphaël Borie, Hans Martin Bosse, Elisabeth Botelho-Nevers, Lila Bouadma, Olivier Bouchaud, Sabelline Bouchez, Damien Bouhour, Kévin Bouiller, Laurence Bouillet, Camile Bouisse, Latsaniphone Bountthasavong, Anne-Sophie Boureau, John Bourke, Maude Bouscambert, Aurore Bousquet, Marielle Boyer-Besseyre, Maria Boylan, Fernando Augusto Bozza, Axelle Braconnier, Cynthia Braga, Timo Brandenburger, Filipa Brás Monteiro, Luca Brazzi, Dorothy Breen, Patrick Breen, David Brewster, Kathy Brickell, Tessa Broadley, Helen Brotherton, Alex Browne, Nicolas Brozzi, Sonja Hjellegjerde Brunvoll, Marjolein Brusse-Keizer, Petra Bryda, Nina Buchtele, Polina Bugaeva, Marielle Buisson, Danilo Buonsenso, Erlina Burhan, Donald Buri, Aidan Burrell, Ingrid G. Bustos, Denis Butnaru, André Cabie, Susana Cabral, Joana Cabrita, Eder Caceres, Cyril Cadoz, Rui Caetano Garcês, Kate Calligy, Jose Andres Calvache, João Camões, Valentine Campana, Paul Campbell, Josie Campisi, Cecilia Canepa, Mireia Cantero, Janice Caoili, Pauline Caraux-Paz, Sheila Cárcel, Filipa Cardoso, Filipe Cardoso, Nelson Cardoso, Sofia Cardoso, Simone Carelli, Nicolas Carlier, Thierry Carmoi, Gayle Carney, Inês Carqueja, Marie-Christine Carret, François Martin Carrier, Ida Carroll, Gail Carson, Maire-Laure Casanova, Mariana Cascão, Siobhan Casey, José Casimiro, Bailey Cassandra, Silvia Castañeda, Nidyanara Castanheira, Guylaine Castor-Alexandre, Ivo Castro, Ana Catarino, François-Xavier Catherine, Paolo Cattaneo, Roberta Cavalin, Giulio Giovanni Cavalli, Alexandros Cavayas, Adrian Ceccato, Masaneh Ceesay, Shelby Cerkovnik, Minerva Cervantes-Gonzalez, Muge Cevik, Anissa Chair, Catherine Chakveatze, Adrienne Chan, Meera Chand, Jean-Marc Chapplain, Charlotte Charpentier, Julie Chas, Muhammad Mobin Chaudry, Jonathan Samuel Chávez Iñiguez, Anjellica Chen, Yih-Sharng Chen, Léo Chenard, Matthew Pellan Cheng, Antoine Cheret, Thibault Chiarabini, Julian Chica, Suresh Kumar Chidambaram, Leong Chin Tho, Catherine Chirouze, Davide Chiumello, Sung-Min Cho, Bernard Cholley, Danoy Chommanam, Marie-Charlotte Chopin, Yock Ping Chow, Ting Soo Chow, Nathaniel Christy, Hiu Jian Chua, Jonathan Chua, Jose Pedro Cidade, José Miguel Cisneros Herreros, Barbara Wanjiru Citarella, Anna Ciullo, Jennifer Clarke, Rolando Claure-Del Granado, Sara Clohisey, Cassidy Codan, Caitriona Cody, Jennifer Coles, Megan Coles, Gwenhaël Colin, Michael Collins, Pamela Combs, Jennifer Connolly, Marie Connor, Anne Conrad, Elaine Conway, Graham S. Cooke, Hugues Cordel, Amanda Corley, Sabine Cornelis, Alexander Daniel Cornet, Arianne Joy Corpuz, Andrea Cortegiani, Grégory Corvaisier, Camille Couffignal, Sandrine Couffin-Cadiergues, Roxane Courtois, Stéphanie Cousse, Juthaporn Cowan, Rachel Cregan, Gloria Crowl, Jonathan Crump, Claudina Cruz, Marc Csete, Ailbhe Cullen, Matthew Cummings, Gerard Curley, Elodie Curlier, Colleen Curran, Paula Custodio, Ana da Silva Filipe, Charlene Da Silveira, Al-Awwab Dabaliz, Andrew Dagens, John Arne Dahl, Darren Dahly, Umberto D'Alessandro, Peter Daley, Zaina Dalloul, Heidi Dalton, Jo Dalton, Seamus Daly, Juliana Damas, Joycelyn Dame, Cammandji Damien, Nick Daneman, Jorge Dantas, Frédérick D'Aragon, Gillian de Loughry, Diego de Mendoza, Etienne De Montmollin, Rafael Freitas de Oliveira França, Ana Isabel de Pinho Oliveira, Rosanna De Rosa, Cristina De Rose, Thushan de Silva, Peter de Vries, Jillian Deacon, David Dean, Alexa Debard, Bianca DeBenedictis, Marie-Pierre Debray, Nathalie DeCastro, William Dechert, Romain Decours, Eve Defous, Isabelle Delacroix, Alexandre Delamou, Eric Delaveuve, Karen Delavigne, Nathalie M. Delfos, Ionna Deligiannis, Andrea Dell'Amore, Christelle Delmas, Pierre Delobel, Corine Delsing, Elisa Demonchy, Emmanuelle Denis, Dominique Deplanque, Pieter Depuydt, Diane Descamps, Mathilde Desvallées, Santi Dewayanti, Pathik Dhangar, Alpha Diallo, Souleymane Taran Diallo, Sylvain Diamantis, André Dias, Fernanda Dias Da Silva, Rodrigo Diaz, Juan Jose Diaz, Priscila Diaz, Bakary K. Dibba, Kévin Didier, Jean-Luc Diehl, Wim Dieperink, Jérôme Dimet, Vincent Dinot, Fara Diop, Alphonsine Diouf, Yael Dishon, Cedric Djadda, Félix Djossou, Annemarie B. Docherty, Helen Doherty, Arjen M. Dondorp, Christl A. Donnelly, Yoann Donohue, Sean Donohue, Peter Doran, Céline Dorival, Eric D'Ortenzio, Yash Doshi, Phouvieng Douangdala, James Joshua Douglas, Renee Douma, Nathalie Dournon, Joanne Downey, Mark Downing, Thomas Drake, Aoife Driscoll, Ibrahim Kwaku Duah, Claudio Duarte Fonseca, Vincent Dubee, François Dubos, Audrey Dubot-Pérès, Alexandre Ducancelle, Toni Duculan, Susanne Dudman, Abhijit Duggal, Paul Dunand, Jake Dunning, Mathilde Duplaix, Emanuele Durante-Mangoni, Lucian Durham, Bertrand Dussol, Juliette Duthoit, Xavier Duval, Anne Margarita Dyrhol-Riise, Sim Choon Ean, Ada Ebo, Marco Echeverria-Villalobos, Michael Edelstein, Siobhan Egan, Linn Margrete Eggesbø, Khadeja Ehzaz, Carla Eira, Mohammed El Sanharawi, Marwan El Sayed, Mohammed Elabid, Mohamed Bashir Elagili, Subbarao Elapavaluru, Mohammad Elbahnasawy, Sohail Elboshra, Brigitte Elharrar, Jacobien Ellerbroek, Merete Ellingjord-Dale, Hamida ELMagrahi, Mohammad Muatasm Elmubark, Loubna Elotmani, Lauren Eloundou, Philippine Eloy, Basma Elshaikhy, Tarek Elshazly, Wafa Elsokni, Aml Ahmed Eltayeb, Iqbal Elyazar, Zarief Kamel Emad, Hussein Embarek, Isabelle Enderle, Tomoyuki Endo, Gervais Eneli, Chan Chee Eng, Ilka Engelmann, Vincent Enouf, Olivier Epaulard, Haneen Esaadi, Mariano Esperatti, Hélène Esperou, Catarina Espírito Santo, Marina Esposito-Farese, Rachel Essaka, Lorinda Essuman, João Estevão, Manuel Etienne, Anna Greti Everding, Mirjam Evers, Isabelle Fabre, Marc Fabre, Ismaila Fadera, Asgad Osman Abdalla Fadlalla, Amna Faheem, Arabella Fahy, Cameron J. Fairfield, Zul Fakar, Komal Fareed, Pedro Faria, Ahmed Farooq, Hanan Fateena, Mohamed Fathi, Salem Fatima, Arie Zainul Fatoni, Karine Faure, Raphaël Favory, Mohamed Fayed, Niamh Feely, Jorge Fernandes, Marília Andreia Fernandes, Susana Fernandes, François-Xavier Ferrand, Eglantine Ferrand Devouge, Joana Ferrão, Mário Ferraz, Benigno Ferreira, Isabel Ferreira, Bernardo Ferreira, Sílvia Ferreira, Nicolas Ferriere, Céline Ficko, Claudia Figueiredo-Mello, William Finlayson, Thomas Flament, Tom Fletcher, Aline-Marie Florence, Letizia Lucia Florio, Brigid Flynn, Deirdre Flynn, Jean Foley, Victor Fomin, Tatiana Fonseca, Patricia Fontela, Karen Forrest, Simon Forsyth, Denise Foster, Giuseppe Foti, Berline Fotso, Erwan Fourn, Robert A. Fowler, Marianne Fraher, Diego Franch-Llasat, Christophe Fraser, John F. Fraser, Marcela Vieira Freire, Ana Freitas Ribeiro, Craig French, Caren Friedrich, Ricardo Fritz, Stéphanie Fry, Nora Fuentes, Masahiro Fukuda, G. Argin, Valérie Gaborieau, Rostane Gaci, Massimo Gagliardi, Jean-Charles Gagnard, Amandine Gagneux-Brunon, Abdou Gai, Sérgio Gaião, Linda Gail Skeie, Adham Mohamed Galal Mohamed Ramadan, Phil Gallagher, Carrol Gamble, Yasmin Gani, Arthur Garan, Rebekha Garcia, Julia Garcia-Diaz, Esteban Garcia-Gallo, Navya Garimella, Denis Garot, Valérie Garrait, Basanta Gauli, Anatoliy Gavrylov, Alexandre Gaymard, Johannes Gebauer, Eva Geraud, Louis Gerbaud Morlaes, Nuno Germano, Malak Ghemmeid, Praveen Kumar Ghisulal, Jade Ghosn, Marco Giani, Tristan Gigante, Elaine Gilroy, Guillermo Giordano, Michelle Girvan, Valérie Gissot, Gezy Giwangkancana, Daniel Glikman, Petr Glybochko, Eric Gnall, Geraldine Goco, François Goehringer, Siri Goepel, Jean-Christophe Goffard, Jin Yi Goh, Brigitta Golács, Jonathan Golob, Kyle Gomez, Joan Gómez-Junyent, Marie Gominet, Alicia Gonzalez, Patricia Gordon, Isabelle Gorenne, Laure Goubert, Cécile Goujard, Tiphaine Goulenok, Margarite Grable, Jeronimo Graf, Edward Wilson Grandin, Pascal Granier, Giacomo Grasselli, Lorenzo Grazioli, Christopher A. Green, Courtney Greene, William Greenhalf, Segolène Greffe, Domenico Luca Grieco, Matthew Griffee, Fiona Griffiths, Ioana Grigoras, Albert Groenendijk, Fassou Mathias Grovogui, Heidi Gruner, Yusing Gu, Jérémie Guedj, Martin Guego, Anne-Marie Guerguerian, Daniela Guerreiro, Romain Guery, Anne Guillaumot, Laurent Guilleminault, Maisa Guimarães de Castro, Thomas Guimard, Marieke Haalboom, Daniel Haber, Ali Hachemi, Abdurrahman Haddud, Nadir Hadri, Wael Hafez, Fakhir Raza Haidri, Fatima Mhd Rida Hajij, Sheeba Hakak, Adam Hall, Matthew Hall, Sophie Halpin, Shaher Hamdan, Abdelhafeez Hamdi, Jawad Hameed, Ansley Hamer, Raph L. Hamers, Rebecca Hamidfar, Bato Hammarström, Naomi Hammond, Terese Hammond, Lim Yuen Han, Matly Hanan, Rashan Haniffa, Kok Wei Hao, Hayley Hardwick, Ewen M. Harrison, Janet Harrison, Samuel Bernard Ekow Harrison, Alan Hartman, Sulieman Hasan, Mohammad Ali Nabil Hasan, Mohd Shahnaz Hasan, Junaid Hashmi, Madiha Hashmi, Amoni Hassan, Ebtisam Hassanin, Muhammad Hayat, Ailbhe Hayes, Leanne Hays, Jan Heerman, Lars Heggelund, Ahmed Helmi, Ross Hendry, Martina Hennessy, Aquiles Rodrigo Henriquez-Trujillo, Maxime Hentzien, Diana Hernandez, Andrew Hershey, Liv Hesstvedt, Astarini Hidayah, Eibhlin Higgins, Rupert Higgins, Samuel Hinton, Hiroaki Hiraiwa, Haider Hirkani, Hikombo Hitoto, Antonia Ho, Yi Bin Ho, Alexandre Hoctin, Isabelle Hoffmann, Wei Han Hoh, Oscar Hoiting, Rebecca Holt, Jan Cato Holter, Peter Horby, Juan Pablo Horcajada, Ikram Houas, Mabrouka Houderi, Catherine L. Hough, Stuart Houltham, Jimmy Ming-Yang Hsu, Jean-Sébastien Hulot, Abby Hurd, Iqbal Hussain, Aliae Mohamed Hussein, Mahmood Hussein, Fatima Ibrahim, Bashir Ibran, Samreen Ijaz, M. Arfan Ikram, Carlos Cañada Illana, Patrick Imbert, Muhammad Imran Ansari, Rana Imran Sikander, Hugo Inácio, Carmen Infante Dominguez, Yun Sii Ing, Mariachiara Ippolito, Vera Irawany, Sarah Isgett, Tiago Isidoro, Nadiah Ismail, Margaux Isnard, Mette Stausland Istre, Junji Itai, Daniel Ivulich, Danielle Jaafar, Salma Jaafoura, Hamza Jaber, Julien Jabot, Clare Jackson, Abubacarr Jagne, Stéphane Jaureguiberry, Denise Jaworsky, Florence Jego, Anilawati Mat Jelani, Synne Jenum, Ruth Jimbo-Sotomayor, Ong Yiaw Joe, Ruth Noemí Jorge García, Silje Bakken Jørgensen, Cédric Joseph, Mark Joseph, Swosti Joshi, Mercé Jourdain, Philippe Jouvet, Anna Jung, Hanna Jung, Dafsah Juzar, Ouifiya Kafif, Florentia Kaguelidou, Neerusha Kaisbain, Thavamany Kaleesvran, Sabina Kali, Karl Trygve Kalleberg, Smaragdi Kalomoiri, Muhammad Aisar Ayadi Kamaluddin, Armand Saloun Kamano, Zul Amali Che Kamaruddin, Nadiah Kamarudin, Kavita Kamineni, Darshana Hewa Kandamby, Kong Yeow Kang, Darakhshan Kanwal, Dyah Kanyawati, Mohamed Karghul, Pratap Karpayah, Todd Karsies, Christiana Kartsonaki, Daisuke Kasugai, Kevin Katz, Christy Kay, Lamees Kayyali, Seán Keating, Pulak Kedia, Andrea Kelly, Aoife Kelly, Claire Kelly, Niamh Kelly, Sadie Kelly, Yvelynne Kelly, Maeve Kelsey, Kalynn Kennon, Sommay Keomany, Maeve Kernan, Younes Kerroumi, Sharma Keshav, Shams Khail, Sarah Khaled, Imrana Khalid, Antoine Khalil, Irfan Khan, Quratul Ain Khan, Sushil Khanal, Abid Khatak, Krish Kherajani, Michelle E. Kho, Denisa Khoo, Ryan Khoo, Saye Khoo, Muhammad Nasir Khoso, Amin Khuwaja, Khor How Kiat, Yuri Kida, Peter Kiiza, Beathe Kiland Granerud, Anders Benjamin Kildal, Jae Burm Kim, Antoine Kimmoun, Detlef Kindgen-Milles, Nobuya Kitamura, Eyrun Floerecke Kjetland Kjetland, Paul Klenerman, Rob Klont, Gry Kloumann Bekken, Stephen R. Knight, Robin Kobbe, Paa Kobina Forson, Chamira Kodippily, Malte Kohns Vasconcelos, Sabin Koirala, Mamoru Komatsu, Franklina Korkor Abebrese, Volkan Korten, Stephanie Kouba, Mohamed Lamine Kourouma, Karifa Kourouma, Arsène Kpangon, Karolina Krawczyk, Ali Kredan, Vinothini Krishnan, Sudhir Krishnan, Oksana Kruglova, Anneli Krund, Pei Xuan Kuan, Ashok Kumar, Deepali Kumar, Ganesh Kumar, Mukesh Kumar, Dinesh Kuriakose, Ethan Kurtzman, Demetrios Kutsogiannis, Galyna Kutsyna, Ama Kwakyewaa Bedu-Addo, Sylvie Kwedi, Konstantinos Kyriakoulis, Marie Lachatre, Marie Lacoste, John G. Laffey, Nadhem Lafhej, Marie Lagrange, Fabrice Laine, Olivier Lairez, Sanjay Lakhey, Marc Lambert, François Lamontagne, Marie Langelot-Richard, Vincent Langlois, Eka Yudha Lantang, Marina Lanza, Cédric Laouénan, Samira Laribi, Delphine Lariviere, Stéphane Lasry, Sakshi Lath, Naveed Latif, Youssef Latifeh, Odile Launay, Didier Laureillard, Yoan Lavie-Badie, Andy Law, Cassie Lawrence, Teresa Lawrence, Minh Le, Clément Le Bihan, Cyril Le Bris, Georges Le Falher, Lucie Le Fevre, Quentin Le Hingrat, Marion Le Maréchal, Soizic Le Mestre, Gwenaël Le Moal, Vincent Le Moing, Hervé Le Nagard, Ema Leal, Marta Leal Santos, Biing Horng Lee, Heng Gee Lee, Su Hwan Lee, James Lee, Jennifer Lee, Todd C. Lee, Yi Lin Lee, Gary Leeming, Bénédicte Lefebvre, Laurent Lefebvre, Benjamin Lefèvre, Sylvie LeGac, Merili-Helen Lehiste, Jean-Daniel Lelievre, François Lellouche, Adrien Lemaignen, Véronique Lemee, Anthony Lemeur, Gretchen Lemmink, Ha Sha Lene, Jenny Lennon, Rafael León, Marc Leone, Tanel Lepik, Quentin Lepiller, François-Xavier Lescure, Olivier Lesens, Mathieu Lesouhaitier, Amy Lester-Grant, Andrew Letizia, Sophie Letrou, Bruno Levy, Yves Levy, Claire Levy-Marchal, Katarzyna Lewandowska, Erwan L'Her, Gianluigi Li Bassi, Janet Liang, Ali Liaquat, Geoffrey Liegeon, Kah Chuan Lim, Wei Shen Lim, Chantre Lima, Bruno Lina, Lim Lina, Andreas Lind, Maja Katherine Lingad, Guillaume Lingas, Sylvie Lion-Daolio, Keibun Liu, Marine Livrozet, Patricia Lizotte, Antonio Loforte, Navy Lolong, Leong Chee Loon, Diogo Lopes, Dalia Lopez-Colon, Anthony L. Loschner, Paul Loubet, Bouchra Loufti, Guillame Louis, Silvia Lourenco, Lara Lovelace-Macon, Lee Lee Low, Marije Lowik, Jia Shyi Loy, Jean Christophe Lucet, Carlos M. Luna, Olguta Lungu, Miles Lunn, Liem Luong, Nestor Luque, Dominique Luton, Olavi Maasikas, Moïse Machado, Sara Machado, Gabriel Macheda, Mustafa Magzoub, Rafael Mahieu, Sophie Mahy, Ana Raquel Maia, Lars S. Maier, Oumou Maiga Ascofare, Mylène Maillet, Thomas Maitre, Nimisha Abdul Majeed, Maximilian Malfertheiner, Nadia Malik, Paddy Mallon, Fernando Maltez, Denis Malvy, Victoria Manda, Laurent Mandelbrot, Frank Manetta, Julie Mankikian, Edmund Manning, Aldric Manuel, Veronika Maráczi, Ceila Maria Sant′Ana Malaque, Flávio Marino, Samuel Markowicz, Ana Marques, Catherine Marquis, Laura Marsh, Brian Marsh, Megan Marshal, John Marshall, Celina Turchi Martelli, Dori-Ann Martin, Emily Martin, Guillaume Martin-Blondel, Alessandra Martinelli, F. Eduardo Martinez, Ignacio Martin-Loeches, Martin Martinot, Alejandro Martín-Quiros, Ana Martins, João Martins, Nuno Martins, Caroline Martins Rego, Gennaro Martucci, Olga Martynenko, Eva Miranda Marwali, Marsilla Marzukie, David Maslove, Sabina Mason, Sobia Masood, Fatma Masoud, Moise Massoma, Palmer Masumbe, Mohd Basri Mat Nor, Moshe Matan, Henrique Mateus Fernandes, Meghena Mathew, Christina Mathew, Mathieu Mattei, Laurence Maulin, Juergen May, Javier Maynar, Mayfong Mayxay, Thierry Mazzoni, Lisa Mc Sweeney, Colin McArthur, Naina McCann, Peter McCanny, Aine McCarthy, Anne McCarthy, Colin McCloskey, Rachael McConnochie, Sherry McDermott, Sarah E. McDonald, Aine McElroy, Samuel McElwee, Natalie McEvoy, Allison McGeer, Kenneth A. McLean, Paul McNally, Bairbre McNicholas, Edel Meaney, Cécile Mear-Passard, Maggie Mechlin, Nastia Medombou, Omar Mehkri, Ferruccio Mele, Luis Melo, Kashif Ali Memon, João João Mendes, Ogechukwu Menkiti, Kusum Menon, France Mentré, Alexander J. Mentzer, Emmanuelle Mercier, Noémie Mercier, Antoine Merckx, Mayka Mergeay-Fabre, Blake Mergler, Laura Merson, António Mesquita, Roberta Meta, Osama Metwally, Agnès Meybeck, Dan Meyer, Alison M. Meynert, Vanina Meysonnier, Mehdi Mezidi, Céline Michelanglei, Isabelle Michelet, Efstathia Mihelis, Vladislav Mihnovit, Duha Milad Abdullah, Jennene Miller, Hugo Miranda-Maldonado, Nor Arisah Misnan, Nik Nur Eliza Mohamed, Nouralsabah Mohamed, Tahira Jamal Mohamed, Alaa Mohamed Ads, Ahmed Reda Mohamed Elsayed Abdelhalim, Libya Mohammed, Shrouk Fawze Mohammed Mostafa, Manahil Omer Abdelrahman Mohammedahmed, Omer Abdullah Mohammedelhassan, Asma Moin, Walaa Mokhtar, Elena Molinos, Brenda Molloy, Mary Mone, Agostinho Monteiro, Claudia Montes, Giorgia Montrucchio, Sarah Moore, Shona C. Moore, Lina Morales Cely, Marwa Morgom, Lucia Moro, Catherine Motherway, Ana Motos, Hugo Mouquet, Clara Mouton Perrot, Julien Moyet, Suleiman Haitham Mualla, Mohamed Muftah, Aisha Kalsoom Mufti, Ng Yong Muh, Mo'nes Muhaisen, Dzawani Muhamad, Jimmy Mullaert, Fredrik Müller, Karl Erik Müller, Daniel Munblit, Syed Muneeb Ali, Nadeem Munir, Laveena Munshi, Aisling Murphy, Patrick Murray, Marlène Murris, Srinivas Murthy, Himed Musaab, Alamin Mustafa, Mus'ab Mustafa, Dana Mustafa, Himasha Muvindi, Dimitra Melia Myrodia, Farah Nadia Mohd-Hanafiah, Behzad Nadjm, Dave Nagpal, Alex Nagrebetsky, Blanka Nagybányai-Nagy, Herwin Nanda Boudoin, Mangala Narasimhan, Nageswaran Narayanan, Prashant Nasa, Rashid Nasim Khan, Ahmad Nasrallah, Adel Gerges Nassif Metri, Alasdair Nazerali-Maitland, Nadège Neant, Holger Neb, Nikita Nekliudov, Matthew Nelder, Erni Nelwan, Raul Neto, Emily Neumann, Wing Yiu Ng, Pauline Yeung Ng, Anthony Nghi, Duc Nguyen, Orna Ni Choileain, Niamh Ni Leathlobhair, Nerissa Niba, Alistair D. Nichol, Prompak Nitayavardhana, Stephanie Nonas, Nurul Amani Mohd Noordin, Nurul Faten Izzati Norharizam, Anita North, Alessandra Notari, Mahdad Noursadeghi, Adam Nowinski, Saad Nseir, Leonard Numfor, Nurnaningsih Nurnaningsih, Dwi Utomo Nusantara, Elsa Nyamankolly, Anders Benteson Nygaard, Fionnuala O. Brien, Annmarie O. Callaghan, Annmarie O'Callaghan, Giovanna Occhipinti, Derbrenn OConnor, Max O'Donnell, Lawrence Ofori-Boadu, Tawnya Ogston, Takayuki Ogura, Tak-Hyuk Oh, Sophie O'Halloran, Katie O'Hearn, Sally-Ann Ohene, Shinichiro Ohshimo, João Oliveira, Larissa Oliveira, Piero L. Olliaro, Cinderella Omar Rageh Elnaggar, Alsarrah Ali Mohammed Omer, Pierre Ondobo, David S.Y. Ong, Jee Yan Ong, Wilna Oosthuyzen, Anne Opavsky, Peter Openshaw, Saijad Orakzai, Claudia Milena Orozco-Chamorro, Jamel Ortoleva, Mohamed Osama Elsayed Soliman, Javier Osatnik, Linda O'Shea, Miriam O'Sullivan, Eman Othman, Siti Zubaidah Othman, Nadia Ouamara, Rachida Ouissa, Micheal Owusu, Ama Akyampomaa Owusu-Asare, Eric Oziol, Maïder Pagadoy, Justine Pages, Amanda Palacios, Massimo Palmarini, Giovanna Panarello, Prasan Kumar Panda, Hem Paneru, Lai Hui Pang, Mauro Panigada, Nathalie Pansu, Aurélie Papadopoulos, Rachael Parke, Melissa Parker, Jérémie Pasquier, Bruno Pastene, Fabian Patauner, Drashti Patel, Mohan Dass Pathmanathan, Luís Patrão, Patricia Patricio, Patricia Patricio, Lisa Patterson, Rajyabardhan Pattnaik, Christelle Paul, Mical Paul, Jorge Paulos, William A. Paxton, Jean-François Payen, Sandra L. Peake, Kalaiarasu Peariasamy, Giles J. Peek, Florent Peelman, Nathan Peiffer-Smadja, Vincent Peigne, Mare Pejkovska, Paolo Pelosi, Ithan D. Peltan, Rui Pereira, Daniel Perez, Thomas Perpoint, Antonio Pesenti, Vincent Pestre, Lenka Petrou, Michele Petrovic, Ventzislava Petrov-Sanchez, Frank Olav Pettersen, Gilles Peytavin, Richard Odame Philips, Ooyanong Phonemixay, Soulichanya Phoutthavong, Michael Piagnerelli, Walter Picard, Olivier Picone, Maria de Piero, Djura Piersma, Carlos Pimentel, Raquel Pinto, Catarina Pires, Lionel Piroth, Ayodhia Pitaloka, Chiara Piubelli, Riinu Pius, Simone Piva, Laurent Plantier, Hon Shen Png, Julien Poissy, Ryadh Pokeerbux, Sergio Poli, Georgios Pollakis, Diane Ponscarme, Diego Bastos Porto, Andra-Maris Post, Douwe F. Postma, Pedro Povoa, Diana Póvoas, Jeff Powis, Sofia Prapa, Viladeth Praphasiri, Sébastien Preau, Christian Prebensen, Jean-Charles Preiser, Anton Prinssen, Mark G. Pritchard, Gamage Dona Dilanthi Priyadarshani, Lucia Proença, Sravya Pudota, Bambang Pujo Semedi, Mathew Pulicken, Peter Puplampu, Gregory Purcell, Luisa Quesada, Vilmaris Quinones-Cardona, Else Quist-Paulsen, Mohammed Quraishi, Fadi Qutishat, Maia Rabaa, Christian Rabaud, Ebenezer Rabindrarajan, Aldo Rafael, Marie Rafiq, Abdelrahman Ragab, Mutia Rahardjani, Arslan Rahat Ullah, Ahmad Kashfi Haji Ab Rahman, Rozanah Abd Rahman, Fernando Rainieri, Giri Shan Rajahram, Pratheema Ramachandran, Nagarajan Ramakrishnan, José Ramalho, Ahmad Afiq Ramli, Blandine Rammaert, Grazielle Viana Ramos, Asim Rana, Rajavardhan Rangappa, Ritika Ranjan, Christophe Rapp, Aasiyah Rashan, Thalha Rashan, Ghulam Rasheed, Menaldi Rasmin, Indrek Rätsep, Cornelius Rau, Tharmini Ravi, Ali Raza, Andre Real, Stanislas Rebaudet, Sarah Redl, Brenda Reeve, Attaur Rehman, Muhammad Osama Rehman Khalid, Dag Henrik Reikvam, Renato Reis, Jordi Rello, Jonathan Remppis, Martine Remy, Hongru Ren, Hanna Renk, Anne-Sophie Resseguier, Matthieu Revest, Oleksa Rewa, Luis Felipe Reyes, Maria Ines Ribeiro, Antonia Ricchiuto, David Richardson, Denise Richardson, Laurent Richier, Siti Nurul Atikah Ahmad Ridzuan, Ana L. Rios, Asgar Rishu, Patrick Rispal, Karine Risso, Maria Angelica Rivera Nuñez, Chiara Robba, André Roberto, Stephanie Roberts, Charles Roberts, David L. Robertson, Olivier Robineau, Anna Roca, Ferran Roche-Campo, Paola Rodari, Simão Rodeia, Bernhard Roessler, Claire Roger, Pierre-Marie Roger, Amanda Rojek, Roberto Roncon-Albuquerque, Mélanie Roriz, Manuel Rosa-Calatrava, Michael Rose, Dorothea Rosenberger, Andrea Rossanese, Matteo Rossetti, Patrick Rossignol, Carine Roy, Benoît Roze, Desy Rusmawatiningtyas, Clark D. Russell, Maeve Ryan, Steffi Ryckaert, Aleksander Rygh Holten, Isabela Saba, Sairah Sadaf, Musharaf Sadat, Valla Sahraei, Abdurraouf Said, Nadia Saidani, Pranya Sakiyalak, Fodé Bangaly Sako, Moamen Salah, Ali Alaa Salah Eldin Mohamed Abbas, Nawal Salahuddin, Leonardo Salazar, Jodat Saleem, Mohammed Saleh Alyasiri, Talat Ahmed Abu Salem, Gabriele Sales, Charlotte Salmon Gandonniere, Hélène Salvator, Dana Samardali, Shaden Samardali, Yehia Samir Shaaban Aly Orabi, Emely Sanchez, Olivier Sanchez, Kizy Sanchez de Oliveira, Angel Sanchez-Miralles, Vanessa Sancho-Shimizu, Gyan Sandhu, Zulfiqar Sandhu, Pierre-François Sandrine, Oana Săndulescu, Marlene Santos, Shirley Sarfo-Mensah, Bruno Sarmento Banheiro, Iam Claire E. Sarmiento, Benjamine Sarton, Ankana Satya, Sree Satyapriya, Rumaisah Satyawati, Egle Saviciute, Yen Tsen Saw, Justin Schaffer, Tjard Schermer, Arnaud Scherpereel, Marion Schneider, János Schnur, Stephan Schroll, Michael Schwameis, Gary Schwartz, Janet T. Scott, James Scott-Brown, Nicholas Sedillot, Tamara Seitz, Jaganathan Selvanayagam, Mageswari Selvarajoo, Malcolm G. Semple, Rasidah Bt Senian, Eric Senneville, Claudia Sepulveda, Filipa Sequeira, Tânia Sequeira, Ary Serpa Neto, Ellen Shadowitz, Syamin Asyraf Shahidan, Hamza Shahla, Laila Shalabi, Haitam Shames, Anuraj Shankar, Shaikh Sharjeel, Pratima Sharma, Catherine A. Shaw, Victoria Shaw, John Robert Sheenan, Dr. Rajesh Mohan Shetty, Rohan Shetty, Mohiuddin Shiekh, Nobuaki Shime, Keiki Shimizu, Sally Shrapnel, Shubha Kalyan Shrestha, Pramesh Sundar Shrestha, Hoi Ping Shum, Nassima Si Mohammed, Ng Yong Siang, Moses Siaw-Frimpong, Jeanne Sibiude, Bountoy Sibounheuang, Nidhal Siddig, Atif Siddiqui, Maqsood Ahmed Siddiqui, Louise Sigfrid, Fatoumata Sillah, Piret Sillaots, Catarina Silva, Maria Joao Silva, Rogério Silva, Benedict Sim Lim Heng, Wai Ching Sin, Dario Sinatti, Mahendra Singh, Punam Singh, Pompini Agustina Sitompul, Karisha Sivam, Vegard Skogen, Sue Smith, Benjamin Smood, Coilin Smyth, Morgane Snacken, Dominic So, Tze Vee Soh, Lene Bergendal Solberg, Joshua Solomon, Tom Solomon, Emily Somers, Agnès Sommet, Myung Jin Song, Rima Song, Tae Song, Jack Song Chia, Arne Søraas, Albert Sotto, Edouard Soum, Ana Chora Sousa, Marta Sousa, Maria Sousa Uva, Vicente Souza-Dantas, Mamadou Saliou Sow, Alexandra Sperry, Elisabetta Spinuzza, B. P. Sanka Ruwan Sri Darshana, Shiranee Sriskandan, Sarah Stabler, Thomas Staudinger, Stephanie-Susanne Stecher, Trude Steinsvik, Ymkje Stienstra, Birgitte Stiksrud, Eva Stolz, Amy Stone, Anca Streinu-Cercel, Adrian Streinu-Cercel, Geoff Strong, Ami Stuart, David Stuart, Richa Su, Decy Subekti, Gabriel Suen, Jacky Y. Suen, Prasanth Sukumar, Asfia Sultana, Charlotte Summers, Dubravka Supic, Deepashankari Suppiah, Magdalena Surovcová, Atie Suwarti, Andrey Svistunov, Sarah Syahrin, Augustina Sylverken, Konstantinos Syrigos, Jaques Sztajnbok, Konstanty Szuldrzynski, Shirin Tabrizi, Fabio S. Taccone, Lysa Tagherset, Shahdattul Mawarni Taib, Sara Taleb, Cheikh Talla, Jelmer Talsma, Renaud Tamisier, Maria Lawrensia Tampubolon, Kim Keat Tan, Yan Chyi Tan, Hiroyuki Tanaka, Taku Tanaka, Hayato Taniguchi, Huda Taqdees, Arshad Taqi, Coralie Tardivon, Yousef Tarek Kamal Mostafa, Ali Tarhabat, Pierre Tattevin, M Azhari Taufik, Hassan Tawfik, Tze Yuan Tee, João Teixeira, Sofia Tejada, Marie-Capucine Tellier, Sze Kye Teoh, Vanessa Teotonio, François Téoulé, Olivier Terrier, Nicolas Terzi, Hubert Tessier-Grenier, Adrian Tey, Alif Adlan Mohd Thabit, Anand Thakur, Zhang Duan Tham, Suvintheran Thangavelu, Elmi Theron, Vincent Thibault, Simon-Djamel Thiberville, Benoît Thill, Jananee Thirumanickam, Niamh Thompson, Shaun Thompson, Emma C. Thomson, David Thomson, Mathew Thorpe, Surain Raaj Thanga Thurai, Ryan S. Thwaites, Paul Tierney, Vadim Tieroshyn, Peter S. Timashev, Jean-François Timsit, Bharath Kumar Tirupakuzhi Vijayaraghavan, Noémie Tissot, Fiona Toal, Jordan Zhien Yang Toh, Maria Toki, Kristian Tonby, Sia Loong Tonnii, Marta Torre, Antoni Torres, Margarida Torres, Rosario Maria Torres Santos-Olmo, Hernando Torres-Zevallos, Aboubacar Tounkara, Michael Towers, Fodé Amara Traoré, Tony Trapani, Cécile Tromeur, Ioannis Trontzas, Tiffany Trouillon, Jeanne Truong, Christelle Tual, Sarah Tubiana, Helen Tuite, Alexis F. Turgeon, Jean-Marie Turmel, Lance C.W. Turtle, Anders Tveita, Pawel Twardowski, Makoto Uchiyama, PG Ishara Udayanga, Andrew Udy, Roman Ullrich, Alberto Uribe, Asad Usman, Effua Usuf, Timothy M. Uyeki, Cristinava Vajdovics, Piero Valentini, Luís Val-Flores, Stijn Van de Velde, Marcel van den Berge, Machteld van der Feltz, Job van der Palen, Paul van der Valk, Nicky Van Der Vekens, Peter Van der Voort, Sylvie Van Der Werf, Laura van Gulik, Jarne Van Hattem, Carolien van Netten, Ilonka van Veen, Noémie Vanel, Henk Vanoverschelde, Michael Varrone, Shoban Raj Vasudayan, Charline Vauchy, Pavan Kumar Vecham, Shaminee Veeran, Aurélie Veislinger, Sebastian Vencken, Sara Ventura, Annelies Verbon, José Ernesto Vidal, César Vieira, Deepak Vijayan, Judit Villar, Pierre-Marc Villeneuve, Andrea Villoldo, Gayatri Vishwanathan, Benoit Visseaux, Hannah Visser, Chiara Vitiello, Manivanh Vongsouvath, Harald Vonkeman, Fanny Vuotto, Suhaila Abdul Wahab, Noor Hidayu Wahab, Nadirah Abdul Wahid, Marina Wainstein, Laura Walsh, Wan Fadzlina Wan Muhd Shukeri, Chih-Hsien Wang, Steve Webb, Jia Wei, Katharina Weil, Tan Pei Wen, Hassi Wesam, Sanne Wesselius, T. Eoin West, Murray Wham, Bryan Whelan, Nicole White, Paul Henri Wicky, Aurélie Wiedemann, Surya Otto Wijaya, Keith Wille, Sue Willems, Bailey Williams, Patricia J. Williams, Virginie Williams, Evert-Jan Wils, Jessica Wittman, Calvin Wong, Xin Ci Wong, Yew Sing Wong, Teck Fung Wong, Natalie Wright, Lim Saio Xian, Ioannis Xynogalas, Siti Rohani Binti Mohd Yakop, Masaki Yamazaki, Elizabeth Yarad, Yazdan Yazdanpanah, Nicholas Yee Liang Hing, Abdelrahman Yehia Mahmoud Abdelaal, Cécile Yelnik, Chian Hui Yeoh, Stephanie Yerkovich, Touxiong Yiaye, Toshiki Yokoyama, Hodane Yonis, Obada Yousif, Saptadi Yuliarto, Akram Zaaqoq, Marion Zabbe, Gustavo E. Zabert, Kai Zacharowski, Masliza Zahid, Maram Zahran, Nor Zaila Binti Zaidan, Maria Zambon, Miguel Zambrano, Alberto Zanella, Nurul Zaynah, Hiba Zayyad, Alexander Zoufaly, David Zucman

**Affiliations:** s Mazankowski Heart Institute, Canada; a ISARIC, Pandemic Sciences Institute, University of Oxford, Oxford, UK; b MRC Population Health Research Unit, Clinical Trials Service Unit and Epidemiological Studies Unit, Nuffield Department of Population Health, University of Oxford, Oxford, UK; c Universidad de La Sabana, Chía, Colombia; d Clínica Universidad de La Sabana, Chía, Colombia; e Big Data Institute, Nuffield Department of Medicine, University of Oxford, Oxford, UK; f Competence Centre for Methodology and Statistics, Luxembourg Institute of Health, Strassen, Luxembourg; g Nuffield Department of Clinical Neurosciences, University of Oxford, Oxford, UK; h Department of Critical Care Medicine, Faculty of Medicine and Dentistry, The University of Alberta, Edmonton, Alberta, Canada; i Department of Intensive Care, Franciscus Gasthuis & Vlietland, Rotterdam, the Netherlands; j Department of Internal Medicine, Hospital Curry Cabral, Centro Hospitalar Universitário de Lisboa Central, Lisbon, Portugal; k Department of Critical Care Medicine, Apollo Hospitals, Chennai, India and The George Institute for Global Health, New Delhi, India; l AIIMS, Rishikesh, India; m Department of Intensive Care Medicine, Multidisciplinary Intensive Care Research Organization (MICRO), St James' Hospital, Dublin, Ireland; n Department of Emergency and Critical Care Medicine, Graduate School of Biomedical and Health Sciences, Hiroshima University, Hiroshima, Japan; o Department of Anesthesiology and Intensive Therapy, Saiful Anwar General Hospital, Brawijaya University, Malang, East Java, Indonesia; p Vall d'Hebrón Institute Research, Barcelona, Spain; q CHU Nîmes, Nîmes, France; r Royal Melbourne Hospital, Melbourne, Australia

**Keywords:** COVID-19, Non-respiratory symptoms, Respiratory symptoms, Risk factors, Mortality

## Abstract

**Background:**

COVID-19 is primarily known as a respiratory illness; however, many patients present to hospital without respiratory symptoms. The association between non-respiratory presentations of COVID-19 and outcomes remains unclear. We investigated risk factors and clinical outcomes in patients with no respiratory symptoms (NRS) and respiratory symptoms (RS) at hospital admission.

**Methods:**

This study describes clinical features, physiological parameters, and outcomes of hospitalised COVID-19 patients, stratified by the presence or absence of respiratory symptoms at hospital admission. RS patients had one or more of: cough, shortness of breath, sore throat, runny nose or wheezing; while NRS patients did not.

**Results:**

Of 178,640 patients in the study, 86.4 % presented with RS, while 13.6 % had NRS. NRS patients were older (median age: NRS: 74 vs RS: 65) and less likely to be admitted to the ICU (NRS: 36.7 % vs RS: 37.5 %). NRS patients had a higher crude in-hospital case-fatality ratio (NRS 41.1 % vs. RS 32.0 %), but a lower risk of death after adjusting for confounders (HR 0.88 [0.83–0.93]).

**Conclusion:**

Approximately one in seven COVID-19 patients presented at hospital admission without respiratory symptoms. These patients were older, had lower ICU admission rates, and had a lower risk of in-hospital mortality after adjusting for confounders.

## 1 Background

Throughout the COVID-19 pandemic, clinical presentation and outcomes have evolved along with virus variants, knowledge of the disease, and levels of care management [1,2]. Early into the pandemic, COVID-19 was predominantly described and managed as a respiratory illness [[3], [4], [5]]. Meanwhile, evidence has accumulated that SARS-CoV-2 infection induces multisystem injury [[6], [7], [8]], affecting cardiovascular, neurological, gastrointestinal, cutaneous, endocrine, renal, musculoskeletal and haematological systems [[8], [9], [10]].

One of the first public health measures to contain transmission of SARS-CoV-2 was identifying febrile patients with respiratory symptoms (RS) and isolating them until laboratory diagnosis was confirmed [11]. However, a proportion of patients with COVID-19 present with no respiratory symptoms (NRS) [12]. A large proportion of COVID-19 patients require in-hospital treatment and have at least one extrapulmonary manifestation during their acute infection [[13], [14], [15], [16]]. However, the clinical outcomes and factors associated with non-respiratory presentations have not been explored systematically [14].

This study attempted to bridge this knowledge gap by characterising the risk factors and clinical outcomes of patients admitted to the hospital with NRS and RS using the ISARIC-WHO database. We hypothesise that the presumed multisystem involvement in patients with NRS is associated with poor prognosis. This information can be relevant to optimise case management and provide helpful information to clinicians treating patients with COVID-19.

## 2 Methods

We used the International Severe Acute Respiratory and Emerging Infection Consortium (ISARIC) - World Health Organization (WHO) Clinical Characterisation Protocol (CCP) for Severe Emerging Infections prospective observational data collection platform for hospitalised patients [17]. Participating sites collected the data prospectively using the ISARIC case report forms (CRFs) built on Research Electronic Data Capture (REDCap, version 8.11.11; Vanderbilt University, Nashville, TN, USA), hosted by the University of Oxford (Oxford, UK). Data were also collected on local databases in other settings and submitted for harmonisation and storage at the University of Oxford. Data were converted to Study Data Tabulation Model standards (version 1.7; Clinical Data Interchange Standards Consortium, Austin, TX, USA) to integrate data collected on locally hosted databases with data collected on the ISARIC database. All investigators retain full rights to their data. The protocol, CRFs, and study information are available on the ISARIC website (https://isaric.org/).

The ISARIC-WHO CCP was approved by the WHO ethics review committee (RPC571 and RPC572). Local ethics approval was obtained for each participating country and site according to local requirements.

### 2.1 Study population

We included patients admitted to the hospital between 30^th^ January 2020 and 30^th^ December 2022 with clinically diagnosed (i.e., symptoms and findings of SARS-CoV-2 pneumonia seen in thoracic diagnostic images) or laboratory-confirmed (i.e., positive reverse transcription polymerase chain reaction) SARS-CoV2 infection according to American Thoracic Society and Infectious Disease Society of America (ATS/IDSA) COVID-19 guidelines [18]. Patients with data on the type of oxygen supplementation status received at any time during their hospitalisation and data on the presence or absence of respiratory symptoms during the first 24 h of admission were included in the study. We excluded patients with missing age or sex, those with missing or unknown respiratory symptoms, and those with missing or negative SARS-CoV-2 status. Sex was defined as the sex assigned at birth and was categorised into male or female.

### 2.2 Variables and measurement

The following variables were included in the analysis: age, sex, comorbidities, complications, country of recruitment and its region according to the World Bank criteria (https://data.worldbank.org/country), vital signs during the first 24 h of admission, treatments, and clinical outcome, that is, in-patient death, and loss to follow up. The key outcome of interest was in-hospital mortality. Patients presenting with one or more symptoms of cough, shortness of breath, sore throat, runny nose or wheezing at the time of hospital admission, irrespective of other symptoms, were classified in the RS group. Regardless of other symptoms, patients not presenting with these respiratory symptoms were classified in the NRS group. Patients who were lost follow-up (i.e., transferred to another hospital or receiving ongoing care) were not considered for fatal outcomes analyses.

### 2.3 Statistical methods

We used descriptive statistics to summarise patient demographics and baseline characteristics. For continuous variables, characteristics were reported as medians and interquartile ranges (IQRs). For categorical variables, counts and percentages were reported. Patient characteristics were compared between the NRS and RS patient groups.

The administration of oxygen therapy at any time during hospitalisation by oxygen delivery methods – basic oxygen therapy, a high-flow nasal cannula (HFNC), non-invasive ventilation (NIV), invasive mechanical ventilation (IMV), and extracorporeal membrane oxygenation (ECMO) – was compared between the NRS and RS patient groups. The overall baseline median (IQR) oxygen saturation (SpO_2_) levels, stratified by age groups, were also compared between the two groups.

We used the Cox proportional hazards model after testing for proportional hazards in the survival analysis to assess the associations of non-respiratory symptoms with the hazard of death. We assessed the proportional hazards assumption using scaled Schoenfeld residuals. Hazard ratios (HRs) and 95 % CIs were estimated for the entire hospitalisation duration and restricted to a shorter hospitalisation duration of 7 and 14 days. Models were adjusted for age (in ten-year age bands), sex, all comorbidities and risk factors, and stratified by country. We grouped countries with less than 50 individuals into a single category.

Comorbidities and risk factors included HIV/AIDS, asthma, cardiac disease, chronic kidney disease, chronic neurological disorder, chronic pulmonary disease, dementia, diabetes, hypertension, liver disease, malignant neoplasm, malnutrition, obesity, smoking, transplantation, rheumatologic disorder and immunosuppression. Immunosuppression was defined according to specific criteria outlined in the case record form for patients who had (i) Pre-admission medication including immunosuppressants such as oral corticosteroids (excluding low-dose hydrocortisone); (ii) People identified as part of clinically extremely vulnerable groups; (iii) People who underwent bone marrow or stem cell transplants within the previous 6 months or were currently under immunosuppression medication; and (iv) People receiving immunosuppressive therapies sufficient to significantly increase risk of infection.

Patients were censored if they were lost to follow-up, which in our dataset could mean they were transferred to another facility or were receiving ongoing care at the time of most recent data collection. Time from symptom onset to time of death or censoring (time to last known to be alive), whichever occurred earlier, was used as the timescale. Patients were considered at risk from symptom onset or admission, whichever occurred later. For all outcomes, censoring times of discharged patients were modified and set to be equal to the maximum time to censoring/event (to account for informative censoring). All statistical analyses were performed using the R statistical programming language, version 4.0.2, and packages *survival, ggplot2*, and *finalfit.*

## 3 Results

**Fig. 1:**
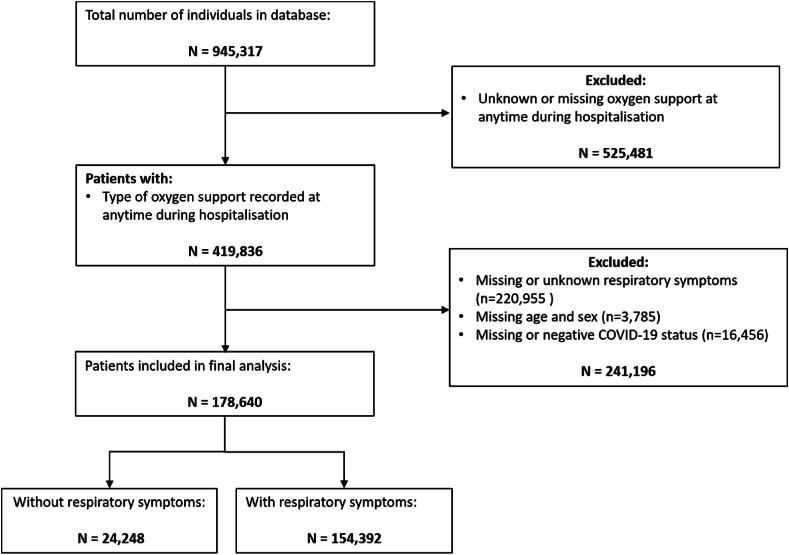
Flow diagram for the study showing the number of patients included in the analysis.

We included a total of 178,640 patients (Fig. 1) from 66 countries.

**Fig. A.1:**
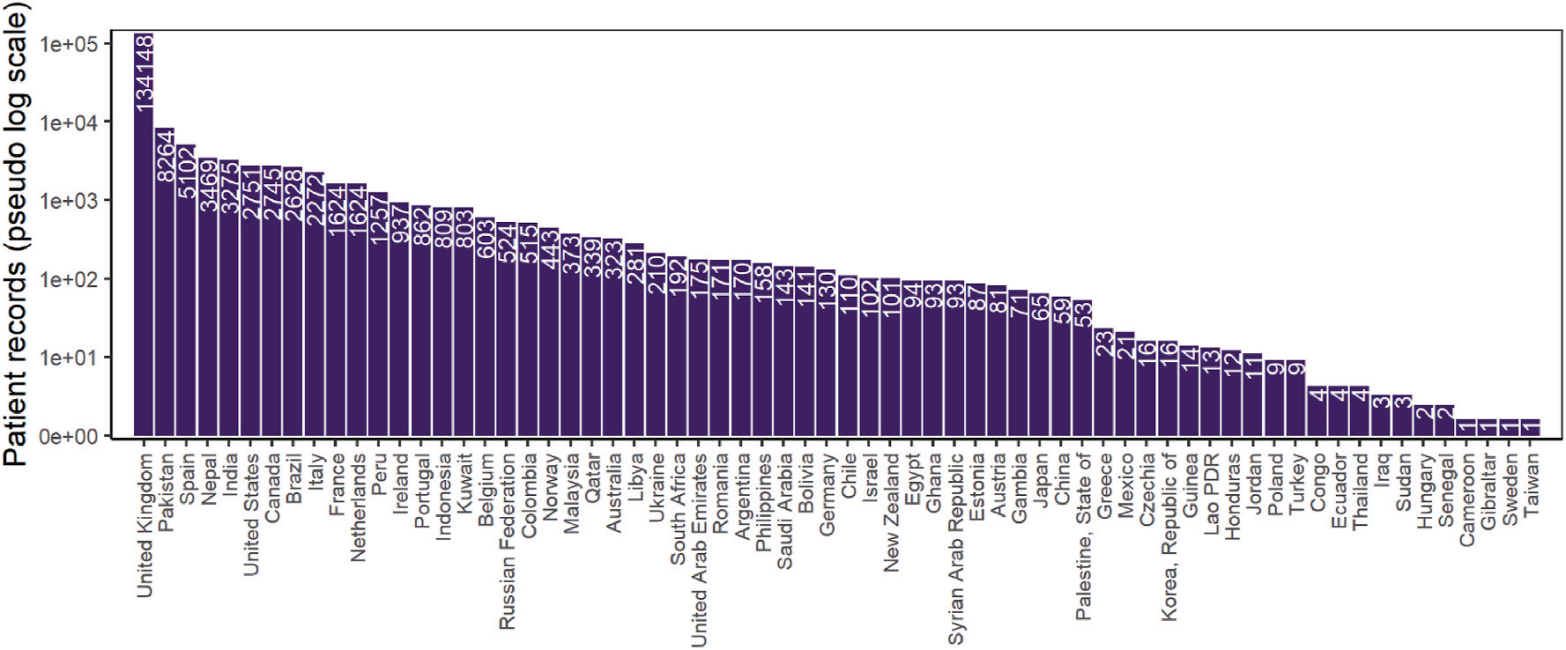
Countries included in the analysis.

Most of the patients were from high-income countries (HIC) (87.1 % [155,648/178,640] and the remainder from low-to-middle-income countries (LMIC) (12.9 % [22,992/178,640]) (Table 1). The countries that contributed the majority of the data were the United Kingdom (75.1 % [134,148/178,640]), Pakistan (4.6 % [8264/178,640]), and Spain 2.9 % [5102/178,640]) (Table A.1; Fig. A.1).

**Table 1:** Baseline characteristics of patients, stratified by respiratory symptoms at hospital admission.

Characteristic	NRS		RS		Total Cohort		*p-value*
	Value (%)	N	Value (%)	N	Value (%)	N	
**Sex, *n (%)***							
Female	10,689 (44.1)	24,248	60,807 (39.4)	154,392	71,496 (40.0)	178,640	<0.001
Male	13,559 (55.9)	24,248	93,585 (60.6)	154,392	107,144 (60.0)	178,640	
**Age, overall, *Median (IQR)***	74 (60–84)	24,248	65.0 (53–77)	154,392	67 (54–79)	178,640	<0.001
**Age, age-groups, *n (%)***							
0 - 19	467 (1.9)	24,248	1406 (0.9)	154,392	1873 (1.0)	178,640	<0.001
20 - 39	1453 (6.0)	24,248	12,502 (8.1)	154,392	13,955 (7.8)	178,640	
40 - 59	4035 (16.6)	24,248	44,121 (28.6)	154,392	48,156 (27.0)	178,640	
60 - 79	9451 (39.0)	24,248	63,895 (41.4)	154,392	73,346 (41.1)	178,640	
>80	8842 (36.5)	24,248	32,468 (21.0)	154,392	41,310 (23.1)	178,640	
**Region, *n (%)***							
East Asia & Pacific	289 (1.2)	24,248	1633 (1.1)	154,392	1922 (1.1)	178,640	<0.001
Europe & Central Asia	18,606 (76.7)	24,248	130,273 (84.4)	154,392	148,879 (83.3)	178,640	
Latin America & Caribbean	441 (1.8)	24,248	4417 (2.9)	154,392	4858 (2.7)	178,640	
Middle East & North Africa	143 (0.6)	24,248	1954 (1.3)	154,392	2097 (1.2)	178,640	
North America	399 (1.6)	24,248	5097 (3.3)	154,392	5496 (3.1)	178,640	
South Asia	4262 (17.6)	24,248	10,746 (7.0)	154,392	15,008 (8.4)	178,640	
Sub-Saharan Africa	108 (0.4)	24,248	272 (0.2)	154,392	380 (0.2)	178,640	
**Income stratification, *n (%)***							
HIC	19,129 (78.9)	24,248	136,519 (88.4)	154,392	155,648 (87.1)	178,640	<0.001
LMIC	5119 (21.1)	24,248	17,873 (11.6)	154,392	22,992 (12.9)	178,640	
**Treatments, *n (%)***							
Vasopressors/Inotropes	2131 (9.0)	23,711	22,433 (15.2)	147,107	24,564 (14.4)	170,818	<0.001
Corticosteroids	10,962 (46.6)	23,535	103,551 (69.4)	149,243	114,513 (66.3)	172,778	<0.001
Intensive care unit	8752 (36.7)	23,834	56,726 (37.5)	151,224	65,478 (37.4)	175,058	0.019
**Comorbidities, *n (%)***							
HIV/AIDS	71 (0.3)	22,918	647 (0.5)	134,185	718 (0.5)	157,103	<0.001
Asthma	2002 (8.5)	23,648	20,164 (14.1)	143,489	22,166 (13.3)	167,137	<0.001
Cardiac disease	7318 (30.7)	23,830	36,621 (25.1)	145,976	43,939 (25.9)	169,806	<0.001
Chronic kidney disease	4102 (17.3)	23,652	19,039 (13.3)	143,689	23,141 (13.8)	167,341	<0.001
Chronic neurological disorder	3255 (13.8)	23,606	12,839 (9.0)	143,361	16,094 (9.6)	166,967	<0.001
Chronic pulmonary disease	2943 (12.4)	23,814	24,362 (16.7)	145,618	27,305 (16.1)	169,432	<0.001
Dementia	3252 (14.0)	23,264	10,632 (7.5)	141,130	13,884 (8.4)	164,394	<0.001
Diabetes	6710 (28.5)	23,576	42,116 (29.3)	143,801	48,826 (29.2)	167,377	**0.01**
Hypertension	10,976 (50.0)	21,947	60,932 (47.4)	128,466	71,908 (47.8)	150,413	<0.001
Immunosuppression	377 (3.1)	12,229	2826 (4.2)	66,685	3203 (4.1)	78,914	<0.001
Liver disease	928 (3.9)	23,924	4257 (2.9)	147,818	5185 (3.0)	171,742	<0.001
Malignant neoplasm	2675 (11.2)	23,805	11,995 (8.3)	145,086	14,670 (8.7)	168,891	<0.001
Malnutrition	616 (2.7)	22,465	2110 (1.6)	133,873	2726 (1.7)	156,338	<0.001
Obesity	1989 (9.2)	21,707	24,790 (19.4)	127,711	26,779 (17.9)	149,418	<0.001
Smoking	4639 (47.3)	9801	37,934 (45.0)	84,346	42,573 (45.2)	94,147	<0.001
Transplantation	149 (1.2)	12,485	1045 (1.5)	69,565	1194 (1.5)	82,050	**0.009**
Rheumatologic disorder	2761 (11.8)	23,413	14,074 (10.0)	140,755	16,835 (10.3)	164,168	<0.001
**Complications, *n (%)***							
Acute Kidney injury	4133 (18.1)	22,895	25,983 (18.8)	138,315	30,116 (18.7)	161,210	0.009
ARDS	1999 (8.8)	22,756	29,751 (21.8)	136,343	31,750 (20.0)	159,099	<0.001
Coagulation Disorder	701 (3.1)	22,658	6941 (5.2)	134,683	7642 (4.9)	157,341	<0.001
Deep Vein Thrombosis	118 (1.0)	11,730	740 (1.0)	73,346	858 (1.0)	85,076	**1.000**
Hyperglycaemia	1932 (8.6)	22,577	22,332 (16.6)	134,414	24,264 (15.5)	156,991	<0.001
Cardiovascular Events	550 (2.4)	22,609	3913 (2.8)	139,585	4463 (2.8)	162,194	**0.002**
Pancreatitis	186 (0.8)	22,901	415 (0.3)	137,720	601 (0.4)	160,621	<0.001
Pleural Effusion	1388 (6.1)	22,739	8992 (6.6)	135,803	10,380 (6.5)	158,542	**0.004**
Pneumothorax	222 (1.0)	22,771	3094 (2.3)	136,166	3316 (2.1)	158,937	<0.001
Pulmonary Embolism	379 (2.2)	17,030	4653 (4.8)	96,543	5032 (4.4)	113,573	<0.001
**Clinical outcomes, *n (%)***							
Loss to follow up	4484 (18.6)	24,062	15,190 (10.1)	150,944	19,674 (11.2)	175,006	<0.001
In-Hospital Mortality	8052 (41.1)	19,578	44,516 (32.0)	139,202	52,568 (33.8)	158,780	

Bold p values indicate no statistical significance.

HIC* = High-income country; LMIC** = Low-to-middle income country; ARDS*** = acute respiratory distress syndrome.

The study population included predominantly males (60.0 % [107,144/178,640]). The overall median (IQR) age was 67 (54–79) years (Table 1), with 41.1 % [73,346/178,640] of patients aged between 60 and 79 years. The most frequent comorbidities and risk factors were hypertension (47.8 % [71,908/150,413]), smoking (45.2 % [42,573/94,147]), diabetes (29.2 % [48,826/167,377]), and cardiac disease (25.9 % [43,939/169,806]) (Table 1). The most frequent complications following admission were acute respiratory distress syndrome (ARDS) (20.0 % [31,750/159,099]) and acute kidney injury (AKI) (18.7 % [30,116/161,210]).

At hospital admission, 13.6 % [24,248/178,640] of patients had no respiratory symptoms. When analysing the cohort per year of the pandemic, the proportion of patients admitted in 2020 with NRS was higher than those admitted in 2021 (2020: 14.6 % [15,320/105,056] vs 2021: 11.1 % [7414/67,054]) (Table A.1).

### 3.1 Clinical characteristics of patients with NRS and RS

Compared to RS patients, NRS patients were older, with a median (IQR) age of 74 (60–84) vs 65 (53–77) for RS patients. There were more male than female patients in both NRS and RS groups, and more male patients in the RS than the NRS group (NRS: 55.9 % [13,559/24,248] and RS: 60.6 % [93,585/154,392]) (Table 1).

**Fig. 2:**
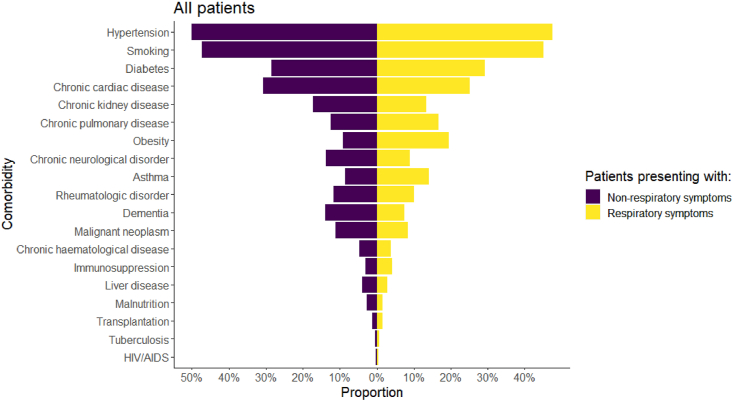
Frequency of comorbidities for all patients, stratified by respiratory symptoms.

**Fig. A.2:**
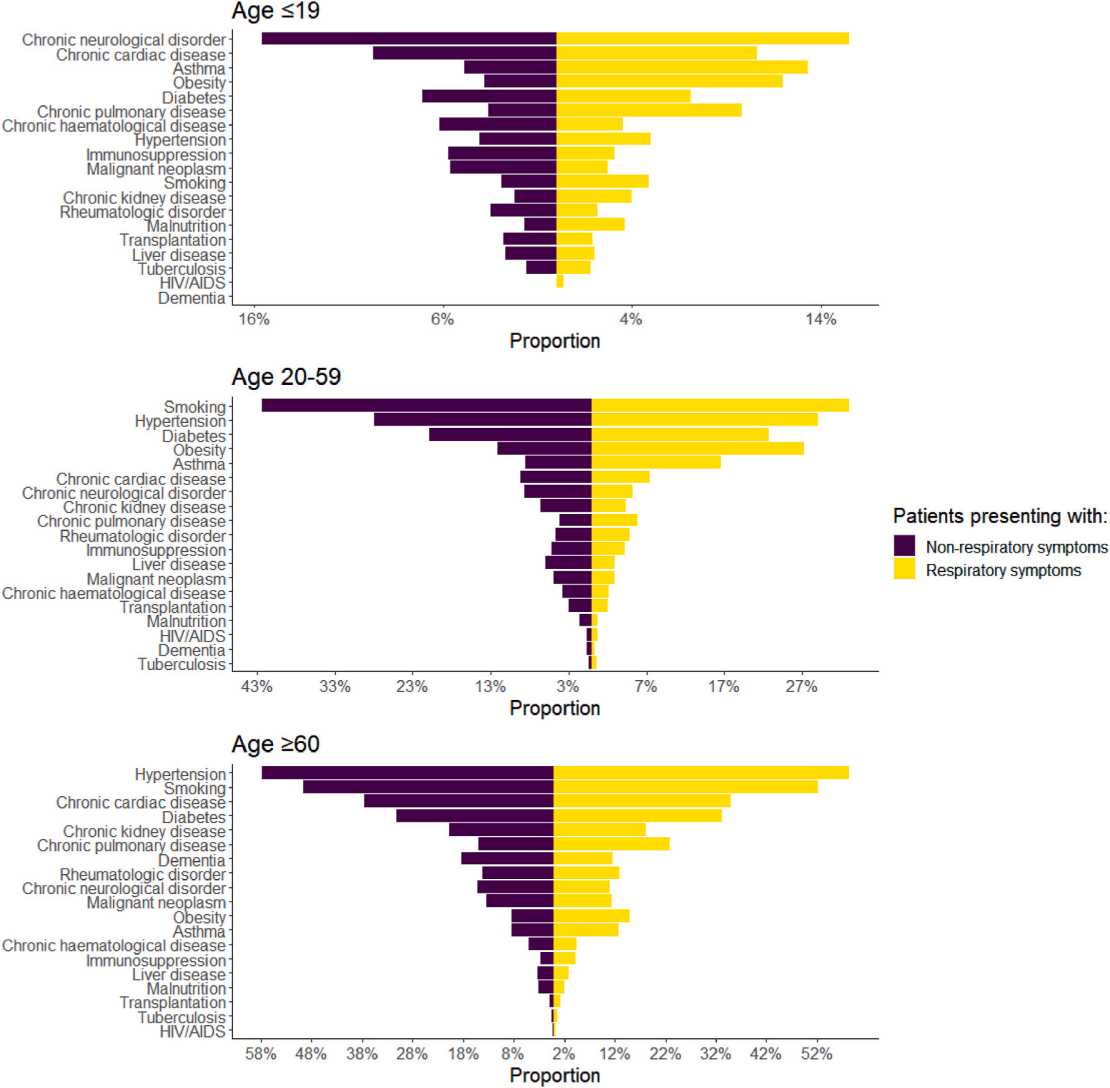
Frequency of comorbidities for different age groups, stratified by respiratory symptoms.

The frequency of some comorbidities and risk factors varied between patients with or without respiratory symptoms: hypertension (NRS: 50.0 % [10,976/21,947] vs RS: 47.4 % [60,392/128,466], *p* < 0.001), smoking (NRS: 47.3 % [4639/9801] vs RS: 45.0 % [37,934/84,346], *p* < 0.001), and cardiac disease (NRS: 30.7 % [7318/23,830] vs RS: 25.1 % [36,621/145,976], *p* < 0.001) were more frequent among patients with NRS; the difference between patients with diabetes was not statistically significant (NRS: 28.5 % [6710/23,576] vs RS: 29.3 % [42,116/143,801], *p* = 0.01). Chronic pulmonary disease and asthma were less frequent among patients with NRS (NRS: 12.4 % [2943/23,814] vs RS: 16.7 % [24,362/145,618], *p* < 0.001; NRS: 8.5 % [2002/23,648] vs RS: 14.1 % [20,164/143,489]), respectively) (Table 1). The distribution of comorbidities and risk factors is presented overall in Fig. 2 and by age groups in Fig. A.2.

### 3.2 Disease severity, systemic complications and outcomes among patients with NRS and RS

During hospitalisation, NRS patients were less likely to be admitted to the ICU (NRS: 36.7 % [8752/23,834] vs RS: 37.5 % [56,726/151,224], *p=*0.019); and were less likely to receive vasopressors (NRS: 9.0 %, [2131/23,711] vs RS: 14.4 %, [22,433/147,107], *p* < 0.001), and corticosteroids (NRS: 46.6 %, [10,962/23,535] vs RS: 69.4 %, [103,551/149,243], *p* < 0.001) (Table 1).

Regarding in-hospital complications, patients with NRS had fewer pulmonary dysfunctions such as ARDS (NRS: 8.8 % [1999/22,756] vs RS: 21.8 % [29,751/136,343], *p* < 0.001); and a significantly lower proportion of coagulation disorders (NRS: 3.1 % [701/22,658] vs RS: 5.2 % [6941/134,683]); hyperglycaemia (NRS: 8.6 % [1932/22,577] vs RS: 16.6 % [22,332/134,414]); pulmonary embolism (NRS: 2.2 % [379/17,030] vs RS: 4.8 % [4653/96,543]); and pneumothorax (NRS: 1.0 % [222/22,771] vs RS: 2.3 % [3094/136,166] (all *p* < 0.001), during their hospitalisation. All systemic complications are reported in Table 1. Finally, patients with NRS had a higher in-hospital mortality rate than patients with RS (NRS: 41.1 % [8052/19,578] vs RS: 32.0 % [44,516/139,202], *p* < 0.001) (Table 1).

**Fig. 3:**
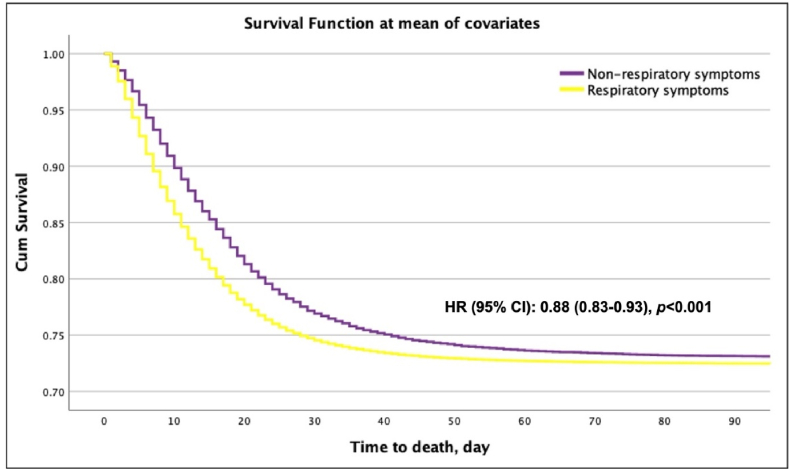
Kaplan—Meier Plot of patients' outcomes stratified by respiratory symptoms.

In the Cox proportional hazards survival analysis, adjusted for age, sex, country, all comorbidities and risk factors, patients with NRS had a lower in-patient mortality risk than patients with RS during their entire hospitalisation (HR [95 % CI] 0.88 (0.83–0.93, p < 0.001) (Table 2; Fig. 3). The in-patient mortality risk remained similar after performing a sensitivity analysis restricted to a shorter hospitalisation duration of 7 and 14 days; however, this was not statistically significant after when restricted to 7 days (Table A.3; Table A.4).

**Table 2:** Hazard ratios (HR) of death by respiratory symptoms group from Cox Proportional Hazards analysis∗.

Variable	HR (95 % CI, p value)	Total Cohort	
**Age group**		**Value n (%)**	**N**
0 - 9	*Reference*		
10 - 19	1.20 (0.30–4.79, p = 0.799)	888 (0.5)	171,828
20 - 29	1.99 (0.69–5.72, p = 0.201)	3682 (2.1)	171,828
30 - 39	1.61 (0.58–4.43, p = 0.358)	9776 (5.7)	171,828
40 - 49	3.21 (1.19–8.63, p = 0.021)	17,032 (9.9)	171,828
50 - 59	5.65 (2.11–15.10, p = 0.001)	28,912 (16.8)	171,828
60 - 69	10.13 (3.80–27.04, p < 0.001)	33,530 (19.5)	171,828
70 - 79	16.25 (6.09–43.36, p < 0.001)	36,848 (21.4)	171,828
80 - 89	22.50 (8.43–60.03, p < 0.001)	30,494 (17.7)	171,828
90 - 99	27.53 (10.30–73.57, p < 0.001)	9558 (5.6)	171,828
>100	38.33 (13.52–108.65, p < 0.001)	241 (0.1)	171,828
**Sex**			
Female	Reference		
Male	1.30 (1.25–1.36, p < 0.001)	102,878 (59.9)	171,828
**Symptoms**			
Respiratory symptoms	Reference		
Non-respiratory symptoms	0.88 (0.83–0.93, p < 0.001)	23,477 (13.7)	171,828
**Comorbidities**∗∗			
HIV/AIDS	0.92 (0.60–1.39, p = 0.685)	620 (0.4)	151,214
Asthma	0.99 (0.93–1.05, p = 0.652)	21,356 (13.3)	160,638
Cardiac disease	1.20 (1.15–1.25, p < 0.001)	42,462 (26.0)	163,233
Chronic kidney disease	1.21 (1.15–1.27, p < 0.001)	22,456 (14.0)	160,837
Chronic neurological disorder	1.12 (1.05–1.19, p < 0.001)	15,541 (9.7)	160,485
Chronic pulmonary disease	1.22 (1.16–1.28, p < 0.001)	26,332 (16.2)	162,872
Dementia	1.25 (1.17–1.33, p < 0.001)	13,585 (8.6)	157,938
Diabetes	1.17 (1.12–1.22, p < 0.001)	47,095 (29.3)	160,869
Hypertension	1.02 (0.98–1.07, p = 0.284)	69,044 (47.9)	144,286
Immunosuppression	1.24 (1.12–1.36, p < 0.001)	3151 (4.1)	77,667
Liver disease	1.33 (1.21–1.48, p < 0.001)	4981 (3.0)	165,130
Malignant neoplasm	1.30 (1.23–1.37, p < 0.001)	14,289 (8.8)	162,359
Malnutrition	1.19 (1.06–1.33, p = 0.003)	2593 (1.7)	151,547
Obesity	1.06 (1.00–1.12, p = 0.039)	25,777 (17.8)	144,740
Rheumatologic disorder	0.96 (0.91–1.02, p = 0.224)	16,169 (10.3)	157,739
Smoking	1.07 (1.02–1.11, p = 0.003)	41,366 (45.4)	91,130
Transplantation	1.34 (1.14–1.57, p < 0.001)	1175 (1.5)	80,847

∗ *Cox proportional hazards model adjusted for age, sex, country, all comorbidities and risk factors*.

∗∗ The reference group for comorbidities is not having the particular comorbidity/risk factor.

**Table 3:** Physiological parameters of patients during the first 24 h, stratified by respiratory symptoms at hospital admission.

Measure	NRS (*n* = 24,248)	RS (*n* = 154,392)	Total Cohort (*n* = 178,640)	*n (%)*
Physiological parameters, *median* (IQR)				
Oxygen saturation *(SpO*_*2*_*)*	96 (93–98)	95 (92–97)	95 (92–97)	80,935 (45.3)
Heart rate *(beats/min)*	87 (76–100)	92 (80–105)	91 (80–104)	162,420 (90.9)
Respiratory rate *(breaths/min)*	20 (18–23)	23 (20–28)	22 (20–28)	159,712 (89.4)
Systolic blood pressure *(mmHg)*	130 (115–145)	129 (115–143)	129 (115–143)	163,314 (91.4)
Diastolic blood pressure *(mmHg)*	73 (65–82)	75 (66–83)	74 (66–83)	163,492 (91.5)
Temperature *(°C)*	36.8 (36.4–37.4)	37.2 (36.7–38)	37.1 (36.6–37.9)	162,450 (90.9)

**Table 4:** Oxygen supplementation at any time during hospitalisation stratified by respiratory symptoms at hospital admission.

Treatment	NRS, *n* (%)	N	RS, *n* (%)	N	Total Cohort, *n* (%)	*p-*value
Basic oxygen therapy	12,771 (52.7)	24,248	66,664 (43.2)	154,392	178,640	<0.001
aAny advanced oxygen	11,477 (47.3)	24,248	87,728 (56.8)	154,392	178,640	<0.001
HFNC	5416 (22.6)	23,962	53,648 (35.7)	150,288	174,250	<0.001
NIV	3491 (14.4)	24,232	45,627 (29.7)	153,695	177,927	<0.001
IMV	6052 (25.0)	24,193	35,840 (23.3)	153,551	177,744	<0.001
ECMO	151 (0.6)	24,126	2263 (1.5)	152,002	176,128	<0.001

HFNC = High Flow nasal cannula; NIV = Non-invasive ventilation.

IMV = Invasive mechanical ventilation; ECMO = Extracorporeal membrane oxygenation.

a Any advanced oxygen = One or more of HFNC, NIV, IMV, ECMO.

Other risk factors associated with the highest increased mortality risks were pre-existing transplantation (HR 1.34 [1.14–1.57], *p* < 0.001), liver disease (1.33 [1.21–1.48], *p* < 0.001), and malignant neoplasm (1.30 [1.23–1.37], p < 0.001) (Table 2).

### 3.3 Oxygen saturation at hospital admission and oxygen supplementation during hospitalisation

**Fig. 4:**
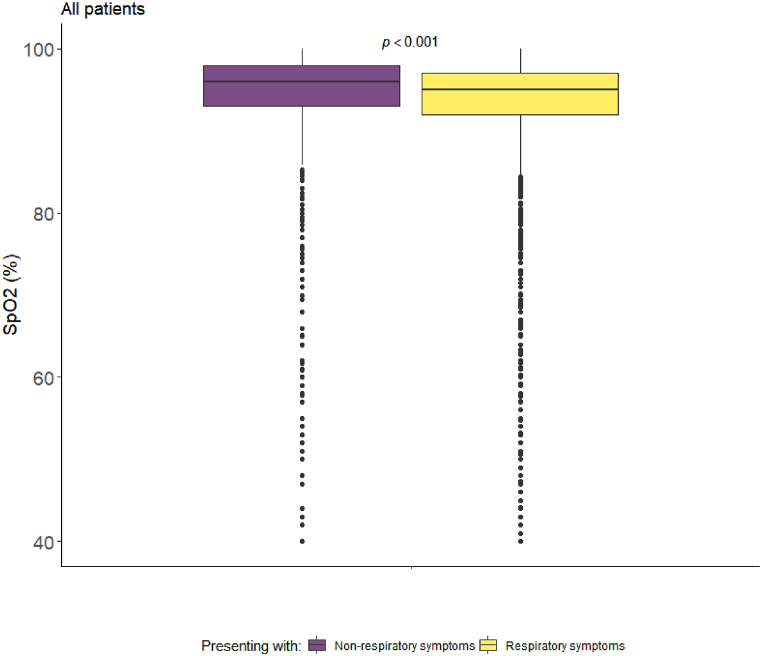
Boxplots of oxygen saturation (SpO_2_) for all patients, stratified by respiratory symptoms.

**Fig. A.3:**
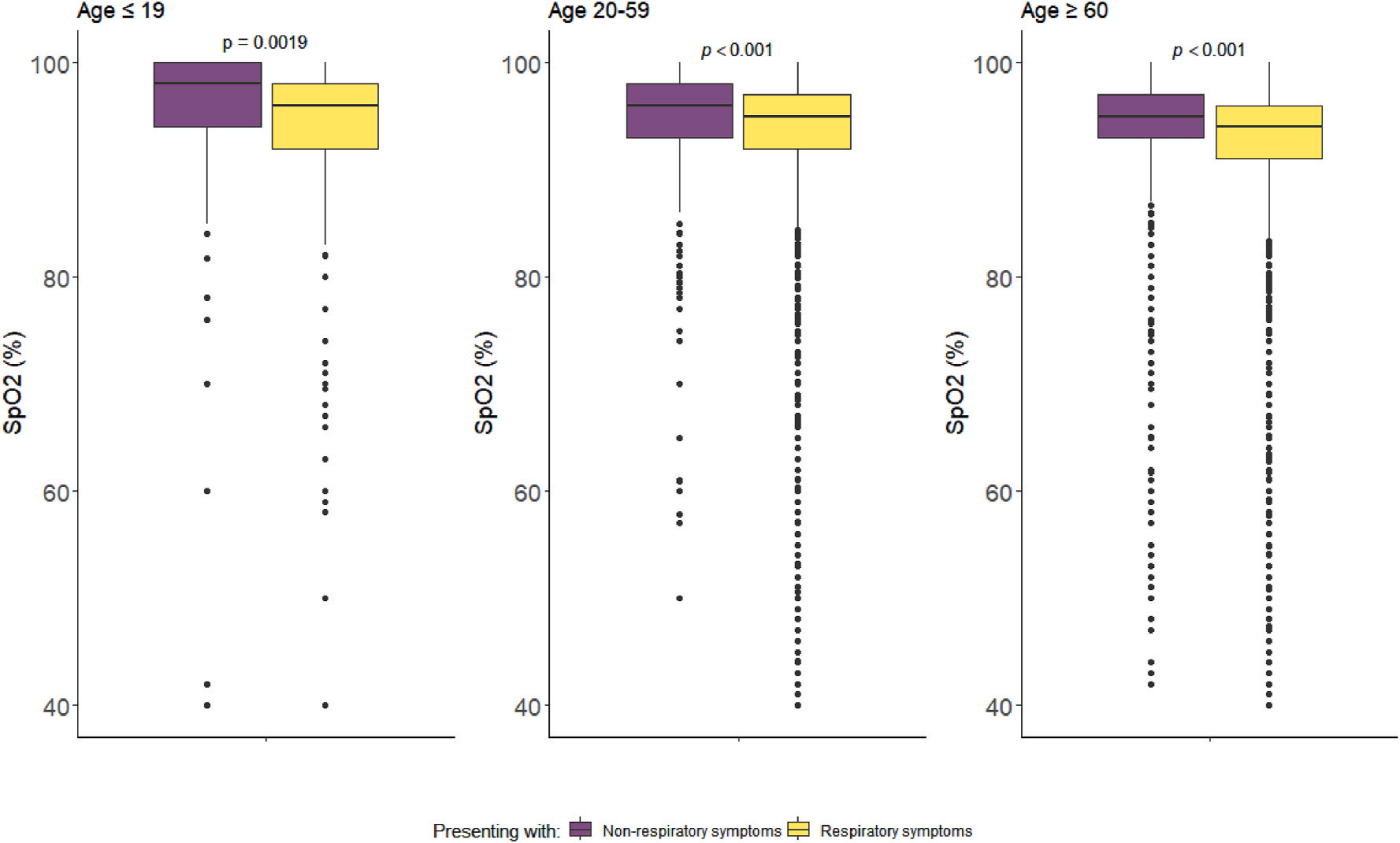
Boxplots of oxygen saturation (SpO2) for different age groups, stratified by respiratory symptoms.

The overall baseline median (IQR) SpO_2_ was higher in NRS patients (NRS: 96 [93–98] vs RS: 95 [92–97], *p* < 0.001 (Table 3; Fig. 4). When stratified by age, NRS patients had higher SpO_2_ levels, and the difference between groups also increased by age (Fig. A.3).

We compared the administration of oxygen therapy at any time during hospitalisation by oxygen delivery methods (Table 4). During hospitalisation, basic oxygen therapy was the most frequent form of oxygen therapy used in NRS patients (52.7 % [12,771/24,248]). Patients with NRS were less likely to receive any advanced oxygen therapy (one or more of HFNC, IMV, NIV or ECMO) compared to RS patients (NRS: 47.3 % [11,477/24,248] and RS: 56.8 % [87,728/154,392], *p* < 0.001). However, NRS patients were more likely to receive IMV compared to RS patients (NRS: 25.0 % [6052/24,193] and RS: 23.3 % [35,840/153,551], *p* < 0.001) (Table 4).

## 4 Discussion

In this large multicentre and prospective cohort, we found that around one in seven hospitalised patients diagnosed with SARS-CoV-2 had no respiratory symptoms of cough, shortness of breath, sore throat, runny nose or wheezing at hospital admission. Compared to those who presented with RS, patients with NRS were older and more likely to suffer from comorbidities other than asthma and chronic pulmonary disease. During hospitalisation, those with NRS were less likely to receive treatment with vasopressors, corticosteroids, and admission to the ICU; however, they developed respiratory failure comparable to those with RS. Notably, the risk for in-hospital mortality was lower in patients with NRS after adjusting for confounders.

COVID-19 has a broad clinical spectrum [10], though its principal manifestation is respiratory [19,20]. Hence, respiratory symptoms have been a critical criterion for identifying SARS-CoV-2 infection [21]. Thus, patients with lung comorbidities have been prioritised during vaccination campaigns for patient care since they are at a higher risk of developing more severe respiratory symptoms [[22], [23], [24]]. This can be attributable to the already dysregulated pulmonary physiology [25,26]. In contrast, at least in the initial phases of COVID-19, patients without apparent respiratory symptoms may be overlooked [8,9,27]. Observational studies have found that almost 30 % of patients manifest atypical symptoms, increasing the risk of misdiagnosis and leading to delays in healthcare, the development of multiorgan failure, and worse clinical outcomes [[28], [29], [30], [31]]. Our results show that most patients with NRS admitted to the hospital required supplementary oxygen at some point during their hospital stay, and almost a third were admitted to ICU, which aligns with prior data [[28], [29], [30], [31]].

One of the main results of our study is that patients with NRS had higher crude in-hospital mortality risk but lower risk than RS patients after adjusting for confounders. Some small prior studies have shown that atypical (most frequently patients with NRS) COVID-19 symptoms are frequent in older patients and are associated with higher mortality [29,32]. Hariyanto et al. and Raymond Pranata et al., in a systematic review and meta-regression, found a significant association of extrapulmonary symptoms, such as delirium with death (OR 1.90 [1.55–2.33], *p* < 0.00001 and 1.50 [1.16, 1.94], *p* = 0.002, respectively). This relationship was not significantly influenced by age, sex, hypertension, diabetes, and dementia [33,34]. Additionally, patients with NRS could develop profound hypoxemia without dyspnoea, called “silent or happy hypoxemia”, which may deteriorate rapidly without warning and has been associated with increased mortality [35]. However, this association remains controversial [[36], [37], [38]].

Early during the pandemic, respiratory symptoms and fever were used to detect patients with possible SARS-CoV-2 infection. However, we found that both patients presenting with and without respiratory symptoms early into the course of COVID-19 could subsequently develop respiratory failure and systemic complications, require oxygen support and die. Targeting patients with respiratory symptoms and/or reduced oxygen saturation will overlook those cases. Jiayi Tan et al., in a systematic review and meta-analysis, found that some public health interventions, such as stroke education campaigns on stroke symptom recognition and intention to call emergency medical services increased the estimated pool risk ratio (RR) for symptoms recognition (RR 1.20) and intention to reach emergency services (RR 1.19) [39].

Our study has strengths and limitations that should be recognised. Firstly, our study population was composed mainly of patients in HICs, which limits the generalisability of these results. Secondly, we do not have complete data on respiratory symptoms, nor extrapulmonary symptoms (i.e., gastrointestinal, cardiac, neurological, among others), during hospitalisation. Therefore, we cannot investigate the association of the progression and impact of respiratory symptoms, nor extrapulmonary symptoms, with outcomes in patients who present with RS or NRS. Moreover, our study had limited data on SARS-CoV-2 variants which restricted our ability to analyse their impact on COVID-19 disease progression. Future studies that incorporate detailed variant data are essential to provide a more in-depth understanding of their impact on COVID-19 patients admitted to hospital with and without respiratory symptoms. Finally, throughout the COVID-19 pandemic, hospitalised patients were treated with a wide range of medications and supportive care protocols, which may bias the factors associated with fatality using observational study methodologies in a fluctuating setting. However, including large numbers of patients over a long period adds to the robustness of our data. To our knowledge, this is one of the largest cohorts comparing patients with NRS and RS globally.

In conclusion, while many COVID-19 patients are hospitalised with respiratory symptoms, about one in seven do not have obvious respiratory symptoms on admission. These NRS patients are usually older and have multiple chronic conditions often unrelated to pulmonary comorbidities. While in the hospital, these patients are less likely to be admitted to the ICU and less likely to receive vasopressors and corticosteroids. About two in five patients may die, but their risk for in-hospital mortality is lower than those presenting with respiratory symptoms after adjusting for confounders. Therefore, more strategies should be implemented to identify patients with COVID-19 and to prevent fatal outcomes in this at-risk population.

## Ethics and consent statement

This observational study required no change to clinical management. The ISARIC-WHO Clinical Characterisation Protocol was approved by the World Health Organization Ethics Review Committee (RPC571 and RPC572 on 25 April 2013). Institutional approval was additionally obtained by participating sites including the South Central Oxford C Research Ethics Committee in England (Ref 13/SC/0149) and the Scotland A Research Ethics Committee (Ref 20/SS/0028) for the United Kingdom, representing the majority of the data. Requirement for consent was waived by the confidentiality advisory group of UK Health Regulations Authority and approved by the sponsor.

Other institutional and national approvals were obtained by participating sites as per local requirements. Regionally appropriate decisions regarding a waiver or requirement of patient consent and/or assent were made by each committee and implemented at the sites.

## Data availability

The data that underpin this analysis are highly detailed clinical data on individuals hospitalised with COVID-19. Due to the sensitive nature of these data and the associated privacy concerns, they are available via a governed data access mechanism following review of a data access committee. Data can be requested via the IDDO COVID-19 Data Sharing Platform (http://www.iddo.org/covid-19). The Data Access Application, Terms of Access and details of the Data Access Committee are available on the website. Briefly, the requirements for access are a request from a qualified researcher working with a legal entity who have a health and/or research remit; a scientifically valid reason for data access which adheres to appropriate ethical principles. The full terms are at: https://www.iddo.org/document/covid-19-data-access-guidelines. A small subset of sites who contributed data to this analysis have not agreed to pooled data sharing as above. In the case of requiring access to these data, please contact the corresponding author in the first instance who will look to facilitate access.

## Funding

This work was made possible by the UK Foreign, Commonwealth and Development Office and Wellcome [215091/Z/18/Z, 222410/Z/21/Z, 225288/Z/22/Z and 220757/Z/20/Z]; the 10.13039/100000865Bill & Melinda Gates Foundation [OPP1209135]; the philanthropic support of the donors to the University of Oxford's COVID-19 Research Response Fund (0009109); grants from the 10.13039/501100000272National Institute for Health Research (NIHR; award CO–CIN-01/DH_/Department of Health/United Kingdom), the 10.13039/501100000265Medical Research Council (MRC; grant MC_PC_19059), and by the 10.13039/501100000272NIHR Health Protection Research Unit (HPRU) in Emerging and Zoonotic Infections at 10.13039/501100000836University of Liverpool in partnership with 10.13039/501100002141Public Health England (PHE), (award 200907), 10.13039/501100000272NIHR HPRU in Respiratory Infections at 10.13039/501100000761Imperial College London with PHE (award 200927), Liverpool Experimental Cancer Medicine Centre (grant C18616/A25153), 10.13039/501100000272NIHR Biomedical Research Centre at 10.13039/501100000761Imperial College London (award ISBRC-1215-20013), and 10.13039/501100000272NIHR Clinical Research Network providing infrastructure support; Cambridge 10.13039/501100000272NIHR Biomedical Research Centre (award NIHR203312); funding from 10.13039/501100000265Medical Research Council (UK Research and Innovation; award number MC_PC_19084) and 10.13039/501100000265Medical Research Council (MC_UU_00031/7); the Comprehensive Local Research Networks (CLRNs) of which PJMO is an 10.13039/501100000272NIHR Senior Investigator (NIHR201385); 10.13039/501100000024CIHR Coronavirus Rapid Research Funding Opportunity OV2170359 and the coordination in Canada by Sunnybrook Research Institute; funding by the Health Research Board of Ireland [CTN-2014-12]; the Rapid European COVID-19 Emergency Response research (RECOVER) [10.13039/100010666H2020 project 101003589] and European Clinical Research Alliance on Infectious Diseases (ECRAID) [965313]; a 10.13039/501100005416Research Council of Norway grant no 312780, and a philanthropic donation from Vivaldi Invest A/S owned by Jon Stephenson von Tetzchner; the South Eastern Norway Health Authority and the Research Council of Norway; 10.13039/501100010767Innovative Medicines Initiative Joint Undertaking under Grant Agreement No. 115523 COMBACTE, resources of which are composed of financial contribution from the European Union's Seventh Framework Programme (FP7/2007–2013) and 10.13039/100013322EFPIA companies, in-kind contribution; the French COVID cohort (NCT04262921) is sponsored by 10.13039/501100015760INSERM and is funded by the REACTing (REsearch & ACtion emergING infectious diseases) consortium and by a grant of the French 10.13039/100009647Ministry of Health (PHRC n°20–0424); Stiftungsfonds zur Förderung der Bekämpfung der Tuberkulose und anderer Lungenkrankheiten of the City of Vienna, Project Number: APCOV22BGM; funding from 10.13039/501100005788Medical University of Vienna, Department of Anaesthesia, Intensive Care Medicine and Pain Medicine; Italian Ministry of Health “Fondi Ricerca corrente–L1P6” to IRCCS Ospedale Sacro Cuore–Don Calabria; Australian Department of Health grant (3273191); Gender Equity Strategic Fund at 10.13039/501100001794University of Queensland, Artificial Intelligence for Pandemics (A14PAN) at 10.13039/501100001794University of Queensland, the 10.13039/501100000923Australian Research Council Centre of Excellence for Engineered Quantum Systems (EQUS, CE170100009), the Prince Charles Hospital Foundation, Australia; Australian Department of Health grant (3273191); Brazil, 10.13039/501100003593National Council for Scientific and Technological Development Scholarship number 303953/2018- 7; the 10.13039/100005815Firland Foundation, Shoreline, Washington, USA; a grant from foundation Bevordering Onderzoek Franciscus; a grant from foundation Bevordering Onderzoek Franciscus; Institute for Clinical Research (ICR), 10.13039/100000002National Institutes of Health (NIH) supported by the Ministry of Health Malaysia; funding from Saisei Mirai/Saisei Pharma, Japan; the U.S. DoD Armed Forces Health Surveillance Division, Global Emerging Infectious Diseases Branch to the U.S Naval Medical Research Unit No. TWO (NAMRU-2) (Work Unit #: P0153_21_N2). These authors would like to thank Vysnova Partners, Inc. for the management of this research project. The Lao-Oxford-Mahosot Hospital-10.13039/100010269Wellcome Trust Research Unit is funded by the 10.13039/100010269Wellcome Trust.

## CRediT authorship contribution statement

**Barbara Wanjiru Citarella:** Writing – review & editing, Writing – original draft, Visualization, Software, Project administration, Methodology, Investigation, Formal analysis, Data curation, Conceptualization. **Christiana Kartsonaki:** Writing – review & editing, Writing – original draft, Software, Methodology, Investigation, Conceptualization, Formal analysis. **Elsa D. Ibáñez-Prada:** Writing – review & editing, Investigation, Writing – original draft. **Bronner P. Gonçalves:** Writing – review & editing, Writing – original draft, Methodology, Investigation. **Joaquin Baruch:** Writing – review & editing, Writing – original draft, Investigation, Methodology. **Martina Escher:** Writing – original draft, Methodology, Investigation, Data curation, Writing – review & editing. **Mark G. Pritchard:** Writing – review & editing, Writing – original draft, Methodology, Investigation, ISARIC Clinical Characterisation Group, Funding acquisition. **Jia Wei:** Writing – review & editing, Writing – original draft, Investigation. **Fred Philippy:** Writing – review & editing, Writing – original draft, Investigation. **Andrew Dagens:** Writing – review & editing, Writing – original draft, Investigation. **Matthew Hall:** Investigation, Writing – original draft, Writing – review & editing. **James Lee:** Investigation, Writing – original draft, Writing – review & editing. **Demetrios James Kutsogiannis:** Writing – review & editing, Writing – original draft, Investigation. **Evert-Jan Wils:** Writing – review & editing, Investigation. **Marília Andreia Fernandes:** Writing – review & editing, Investigation. **Bharath Kumar Tirupakuzhi Vijayaraghavan:** Investigation, Writing – review & editing. **Prasan Kumar Panda:** Writing – review & editing, Investigation. **Ignacio Martin-Loeches:** Writing – review & editing, Investigation. **Shinichiro Ohshimo:** Writing – review & editing, Investigation. **Arie Zainul Fatoni:** Investigation, Writing – review & editing. **Peter Horby:** Conceptualization, Writing – review & editing. **Jake Dunning:** Writing – review & editing, Conceptualization, Investigation, Methodology, Writing – original draft. **Jordi Rello:** Writing – review & editing, Investigation. **Laura Merson:** Writing – original draft, Project administration, Methodology, Investigation, Funding acquisition, Conceptualization. **Amanda Rojek:** Writing – review & editing, Writing – original draft, Investigation, Conceptualization, Methodology. **Michel Vaillant:** Writing – original draft, Methodology, Investigation, Conceptualization, Writing – review & editing. **Piero Olliaro:** Writing – original draft, Methodology, Investigation, Conceptualization, Writing – review & editing. **Luis Felipe Reyes:** Methodology, Writing – original draft, Writing – review & editing, Conceptualization, Investigation.

## Declaration of competing interest

The authors declare the following financial interests/personal relationships which may be considered as potential competing interests:IML declared lectures for Gilead, Thermofisher, MSD; advisory board participation for Fresenius Kabi, Advanz Pharma, Gilead, Accelerate, Merck; and consulting fees for Gilead outside of the submitted work.

## Figures and Tables

**Table A.1:** Demographics of patients, stratified by respiratory symptoms.

Characteristic	NRS Value (%)	RS Value (%)	Total Cohort Value (%) N	
Year of admission				
2020	15,320 (63.4)	89,736 (58.2)	105,056 (58.9)	178,297
2021	7414 (30.7)	59,640 (38.7)	67,054 (37.6)	178,297
2022	1433 (5.9)	4754 (3.1)	6187 (3.5)	178,297
**Country**				
United Kingdom	17,580 (72.5)	116,568 (75.5)	134,148 (75.1)	178,640
Pakistan	1954 (8.1)	6310 (4.1)	8264 (4.6)	178,640
Spain	385 (1.6)	4717 (3.1)	5102 (2.9)	178,640
Nepal	752 (3.1)	2717 (1.8)	3469 (1.9)	178,640
India	1556 (6.4)	1719 (1.1)	3275 (1.8)	178,640
United States	173 (0.7)	2578 (1.7)	2751 (1.5)	178,640
Canada	226 (0.9)	2519 (1.6)	2745 (1.5)	178,640
Brazil	300 (1.2)	2328 (1.5)	2628 (1.5)	178,640
Italy	92 (0.4)	2180 (1.4)	2272 (1.3)	178,640
France	95 (0.4)	1529 (1.0)	1624 (0.9)	178,640
Netherlands	72 (0.3)	1552 (1.0)	1624 (0.9)	178,640
Peru	68 (0.3)	1189 (0.8)	1257 (0.7)	178,640
Ireland	119 (0.5)	818 (0.5)	937 (0.5)	178,640
Portugal	106 (0.4)	756 (0.5)	862 (0.5)	178,640
Indonesia	58 (0.2)	751 (0.5)	809 (0.5)	178,640
Kuwait	32 (0.1)	771 (0.5)	803 (0.4)	178,640
Belgium	70 (0.3)	533 (0.3)	603 (0.3)	178,640
Russian Federation	23 (0.1)	501 (0.3)	524 (0.3)	178,640
Colombia	43 (0.2)	472 (0.3)	515 (0.3)	178,640
Norway	19 (0.1)	424 (0.3)	443 (0.2)	178,640
Malaysia	151 (0.6)	222 (0.1)	373 (0.2)	178,640
Qatar	22 (0.1)	317 (0.2)	339 (0.2)	178,640
Australia	32 (0.1)	291 (0.2)	323 (0.2)	178,640
Libya	11 (0.0)	270 (0.2)	281 (0.2)	178,640
Ukraine	0 (0.0)	210 (0.1)	210 (0.1)	178,640
South Africa	8 (0.0)	184 (0.1)	192 (0.1)	178,640
United Arab Emirates	7 (0.0)	168 (0.1)	175 (0.1)	178,640
Romania	26 (0.1)	145 (0.1)	171 (0.1)	178,640
Argentina	14 (0.1)	156 (0.1)	170 (0.1)	178,640
Philippines	33 (0.1)	125 (0.1)	158 (0.1)	178,640
Saudi Arabia	45 (0.2)	98 (0.1)	143 (0.1)	178,640
Bolivia	4 (0.0)	137 (0.1)	141 (0.1)	178,640
Germany	8 (0.0)	122 (0.1)	130 (0.1)	178,640
Chile	6 (0.0)	104 (0.1)	110 (0.1)	178,640
Israel	20 (0.1)	82 (0.1)	102 (0.1)	178,640
New Zealand	3 (0.0)	98 (0.1)	101 (0.1)	178,640
Egypt	0 (0.0)	94 (0.1)	94 (0.1)	178,640
Ghana	57 (0.2)	36 (0.0)	93 (0.1)	178,640
Syrian Arab Republic	1 (0.0)	92 (0.1)	93 (0.1)	178,640
Estonia	0 (0.0)	87 (0.1)	87 (0.0)	178,640
Austria	4 (0.0)	77 (0.0)	81 (0.0)	178,640
Gambia	40 (0.2)	31 (0.0)	71 (0.0)	178,640
Japan	4 (0.0)	61 (0.0)	65 (0.0)	178,640
China	6 (0.0)	53 (0.0)	59 (0.0)	178,640
Palestine, State of	4 (0.0)	49 (0.0)	53 (0.0)	178,640
Greece	4 (0.0)	19 (0.0)	23 (0.0)	178,640
Mexico	0 (0.0)	21 (0.0)	21 (0.0)	178,640
Czechia	3 (0.0)	13 (0.0)	16 (0.0)	178,640
Korea, Republic of	1 (0.0)	15 (0.0)	16 (0.0)	178,640
Guinea	2 (0.0)	12 (0.0)	14 (0.0)	178,640
Lao PDR	0 (0.0)	13 (0.0)	13 (0.0)	178,640
Honduras	6 (0.0)	6 (0.0)	12 (0.0)	178,640
Jordan	1 (0.0)	10 (0.0)	11 (0.0)	178,640
Poland	0 (0.0)	9 (0.0)	9 (0.0)	178,640
Turkey	0 (0.0)	9 (0.0)	9 (0.0)	178,640
Congo	0 (0.0)	4 (0.0)	4 (0.0)	178,640
Ecuador	0 (0.0)	4 (0.0)	4 (0.0)	178,640
Thailand	0 (0.0)	4 (0.0)	4 (0.0)	178,640
Iraq	0 (0.0)	3 (0.0)	3 (0.0)	178,640
Sudan	0 (0.0)	3 (0.0)	3 (0.0)	178,640
Senegal	1 (0.0)	1 (0.0)	2 (0.0)	178,640
Hungary	0 (0.0)	2 (0.0)	2 (0.0)	178,640
Taiwan	1 (0.0)	0 (0.0)	1 (0.0)	178,640
Cameroon	0 (0.0)	1 (0.0)	1 (0.0)	178,640
Gibraltar	0 (0.0)	1 (0.0)	1 (0.0)	178,640
Sweden	0 (0.0)	1 (0.0)	1 (0.0)	178,640

**Table A.2:** General symptoms at admission, stratified by respiratory symptoms.

Variable	NRS		RS		Total Cohort	
	Value (%)	N	Value (%)	N	Value (%)	N
Conjunctivitis	41 (0.2)	24,090	443 (0.3)	128,763	484 (0.3)	152,853
Ear pain	24 (0.1)	22,449	359 (0.3)	105,267	383 (0.3)	127,716
Headache	928 (3.9)	24,036	15,737 (12.2)	128,518	16,665 (10.9)	152,554
Lost/altered sense of smell	224 (1.0)	21,699	9528 (8.8)	108,566	9752 (7.5)	130,265
Lost/altered sense of taste	318 (1.5)	21,708	11,387 (10.7)	106,209	11,705 (9.2)	127,917
Chest pain	889 (3.7)	24,187	22,020 (16.0)	137,793	22,909 (14.1)	161,980
Abdominal pain	2072 (8.6)	24,121	9188 (6.8)	134,226	11,260 (7.1)	158,347
Joint pain	1571 (6.5)	24,030	27,231 (21.2)	128,510	28,802 (18.9)	152,540
Fatigue	4521 (18.8)	24,028	62,837 (46.5)	135,011	67,358 (42.4)	159,039
Fever	7070 (29.3)	24,130	101,835 (69.0)	147,679	108,905 (63.4)	171,809
Vomiting/nausea	2991 (12.4)	24,185	23,593 (17.0)	138,867	26,584 (16.3)	163,052
Diarrhoea	2085 (8.6)	24,168	25,406 (18.2)	139,405	27,491 (16.8)	163,573
Severe Dehydration	1284 (14.2)	9016	7126 (12.2)	58,293	8410 (12.5)	67,309
Skin rash	452 (1.9)	24,085	2414 (1.8)	131,269	2866 (1.8)	155,354
Bleeding	559 (2.3)	24,124	1735 (1.3)	133,709	2294 (1.5)	157,833
Confusion	5275 (21.9)	24,091	23,117 (16.5)	139,827	28,392 (17.3)	163,918
Seizures	496 (2.1)	24,117	997 (0.7)	136,215	1493 (0.9)	160,332
Lymphadenopathy	62 (0.3)	23,952	607 (0.5)	128,575	669 (0.4)	152,527

**Table A.3:** Hazard ratios (HR) of death by symptoms group from Cox Proportional Hazards analysis^a^, restricted to 7 days hospitalisation

Variable	HR (95 % CI, p value)	Total Cohort	
**Age group**		**Value n (%)**	**N**
0–9	Reference		
10–19	1.21 (0.30–4.85, p = 0.785)	888 (0.5)	171,828
20–29	2.03 (0.71–5.83, p = 0.189)	3682 (2.1)	171,828
30–39	1.63 (0.59–4.49, p = 0.345)	9776 (5.7)	171,828
40–49	3.26 (1.21–8.75, p = 0.019)	17,032 (9.9)	171,828
50–59	5.76 (2.15–15.38, p < 0.001)	28,912 (16.8)	171,828
60–69	10.29 (3.85–27.46, p < 0.001)	33,530 (19.5)	171,828
70–79	16.22 (6.08–43.27, p < 0.001)	36,848 (21.4)	171,828
80–89	22.13 (8.29–59.05, p < 0.001)	30,494 (17.7)	171,828
90–99	26.95 (10.09–72.03, p < 0.001)	9558 (5.6)	171,828
>100	35.96 (12.68–101.97, p < 0.001)	241 (0.1)	171,828
**Sex**			
Female	Reference		
Male	1.29 (1.24–1.35, p < 0.001)	102,878 (59.9)	171,828
**Symptoms**			
Respiratory symptoms	Reference		
Non-respiratory symptoms	0.91 (0.86–0.96, p = 0.001)	23,477 (13.7)	171,828
**Comorbidities**^b^			
HIV/AIDS	0.92 (0.61–1.40, p = 0.696)	620 (0.4)	151,214
Asthma	0.98 (0.93–1.04, p = 0.599)	21,356 (13.3)	160,638
Cardiac disease	1.19 (1.14–1.24, p < 0.001)	42,462 (26.0)	163,233
Chronic kidney disease	1.21 (1.15–1.27, p < 0.001)	22,456 (14.0)	160,837
Chronic neurological disorder	1.11 (1.04–1.18, p = 0.001)	15,541 (9.7)	160,485
Chronic pulmonary disease	1.21 (1.16–1.27, p < 0.001)	26,332 (16.2)	162,872
Dementia	1.23 (1.15–1.31, p < 0.001)	13,585 (8.6)	157,938
Diabetes	1.16 (1.12–1.22, p < 0.001)	47,095 (29.3)	160,869
Hypertension	1.02 (0.98–1.06, p = 0.363)	69,044 (47.9)	144,286
Immunosuppression	1.24 (1.13–1.37, p < 0.001)	3151 (4.1)	77,667
Liver disease	1.34 (1.21–1.49, p < 0.001)	4981 (3.0)	165,130
Malignant neoplasm	1.29 (1.22–1.36, p < 0.001)	14,289 (8.8)	162,359
Malnutrition	1.16 (1.03–1.30, p = 0.011)	2593 (1.7)	151,547
Obesity	1.06 (1.00–1.12, p = 0.043)	25,777 (17.8)	144,740
Rheumatologic disorder	0.96 (0.90–1.02, p = 0.146)	16,169 (10.3)	157,739
Smoking	1.07 (1.02–1.11, p = 0.002)	41,366 (45.4)	91,130
Transplantation	1.34 (1.14–1.57, p < 0.001)	1175 (1.5)	80,847

a *Cox proportional hazards model adjusted for age, sex, country, all comorbidities and risk factors*.

b The reference group for comorbidities is not having the particular comorbidity/risk factor.

**Table A.4:** Hazard ratios (HR) of death by symptoms group from Cox Proportional Hazards analysis^a^, restricted to 14 days hospitalisation

Variable	HR (95 % CI, p value)	Total Cohort	
**Age group**		**Value n (%)**	**N**
0–9	Reference		
10–19	1.20 (0.30–4.80, p = 0.796)	888 (0.5)	171,828
20–29	2.00 (0.70–5.75, p = 0.198)	3682 (2.1)	171,828
30–39	1.61 (0.58–4.44, p = 0.356)	9776 (5.7)	171,828
40–49	3.21 (1.20–8.64, p = 0.021)	17,032 (9.9)	171,828
50–59	5.65 (2.11–15.09, p = 0.001)	28,912 (16.8)	171,828
60–69	10.10 (3.79–26.96, p < 0.001)	33,530 (19.5)	171,828
70–79	16.13 (6.04–43.03, p < 0.001)	36,848 (21.4)	171,828
80–89	22.25 (8.34–59.37, p < 0.001)	30,494 (17.7)	171,828
90–99	27.17 (10.17–72.60, p < 0.001)	9558 (5.6)	171,828
>100	37.90 (13.37–107.46, p < 0.001)	241 (0.1)	171,828
**Sex**			
Female	Reference		
Male	1.30 (1.25–1.35, p < 0.001)	102,878 (59.9)	171,828
**Symptoms**			
Respiratory symptoms	Reference		
Non-respiratory symptoms	0.88 (0.84–0.93, p < 0.001)	23,477 (13.7)	171,828
**Comorbidities**^b^			
HIV/AIDS	0.90 (0.59–1.36, p = 0.609)	620 (0.4)	151,214
Asthma	0.99 (0.93–1.05, p = 0.662)	21356 (13.3)	160638
Cardiac disease	1.20 (1.15–1.25, p < 0.001)	42462 (26.0)	163233
Chronic kidney disease	1.21 (1.15–1.27, p < 0.001)	22456 (14.0)	160837
Chronic neurological disorder	1.12 (1.05–1.19, p = 0.001)	15541 (9.7)	160485
Chronic pulmonary disease	1.22 (1.16–1.28, p < 0.001)	26332 (16.2)	162872
Dementia	1.24 (1.17–1.33, p < 0.001)	13585 (8.6)	157938
Diabetes	1.17 (1.12–1.22, p < 0.001)	47095 (29.3)	160869
Hypertension	1.02 (0.98–1.07, p = 0.282)	69044 (47.9)	144286
Immunosuppression	1.23 (1.12–1.36, p < 0.001)	3151 (4.1)	77667
Liver disease	1.34 (1.21–1.48, p < 0.001)	4981 (3.0)	165130
Malignant neoplasm	1.29 (1.23–1.37, p < 0.001)	14289 (8.8)	162359
Malnutrition	1.17 (1.04–1.31, p = 0.007)	2593 (1.7)	151547
Obesity	1.06 (1.00–1.12, p = 0.051)	25777 (17.8)	144740
Rheumatologic disorder	0.96 (0.91–1.02, p = 0.214)	16169 (10.3)	157739
Smoking	1.07 (1.02–1.11, p = 0.002)	41366 (45.4)	91130
Transplantation	1.34 (1.14–1.58, p < 0.001)	1175 (1.5)	80847

a *Cox proportional hazards model adjusted for age, sex, country, all comorbidities and risk factors*.

b The reference group for comorbidities is not having the particular comorbidity/risk factor.
